# The effects of twenty-one nutrients and phytonutrients on cognitive function: A narrative review

**Published:** 2021-08-04

**Authors:** John E. Lewis, Jillian Poles, Delaney P. Shaw, Elisa Karhu, Sher Ali Khan, Annabel E. Lyons, Susana Barreiro Sacco, H. Reginald McDaniel

**Affiliations:** ^1^Department of Psychiatry and Behavioral Sciences, University of Miami Miller School of Medicine, Miami, FL, USA; ^2^Department of Exercise and Sport Science, University of North Carolina at Chapel Hill, Chapel Hill, NC, USA; ^3^Institute of Human Nutrition, Vagelos College of Physicians and Surgeons, Columbia University, New York, NY, USA; ^4^Department of Medicine, University of Miami Miller School of Medicine, Miami, FL, USA; ^5^Department of Internal Medicine, University of New Mexico Health Sciences Center, Albuquerque, NM, USA; ^6^School of Nursing and Health Studies, University of Miami, Coral Gables, FL, USA; ^7^Department of Internal Medicine, Mount Sinai Medical Center Miami Beach, FL, USA; ^8^Wellness Quest, LLC, Grand Prairie, TX, USA

**Keywords:** Alzheimer’s disease, cognitive function, cognitive impairment, memory, nutrients, phytonutrients

## Abstract

**Background and Aim:**

Brain health is becoming more important to the average person as the number of people with cognitive impairments, such as Alzheimer’s disease (AD), is rising significantly. The current Food and Drug Administration-approved pharmacotherapeutics for dementia neither cure nor halt cognitive decline; they just delay the worsening cognitive impairment. This narrative review summarizes the effects of nutrients and phytonutrients on cognitive function.

**Methods:**

A comprehensive literature search of PubMed was performed to find clinical trials in humans that assessed the effects of nutrients and phytonutrients on cognitive function published in English between 2000 and 2021. Six independent reviewers evaluated the articles for inclusion in this review.

**Results:**

Ninety-six articles were summarized in this narrative review. In total 21 categories of nutrients and phytonutrients were included, i.e., α-lipoic acid, *Bacopa monnieri*, B vitamins, cholinergic precursors, vitamin D, vitamin E, *Ginkgo biloba*, ginseng, lion’s mane mushroom, N-acetyl cysteine, omega-3 fatty acids, aloe polysaccharides, *Rhodiola rosea*, rosemary, saffron, tart cherries, turmeric, wild yam, *Withania somnifera*, xanthines, and zinc. Particular noteworthy effects on cognition included memory, recollection, attention, intelligence, vocabulary, recognition, response inhibition, arousal, performance enhancement, planning, creative thinking, reaction time, vigilance, task switching, orientation to time, place, and person, reading, writing, comprehension, accuracy, learning, information processing speed, executive function, mental flexibility, daily functioning, decrease in mental fatigue, and freedom from distractibility. Some nutrients and phytonutrients also improved mood and contentedness and reduced anxiety and the need for caregiving. These effects are not completely consistent or ubiquitous across all patient populations or health statuses. Adverse effects were minimal or nonexistent.

**Conclusion:**

Due to the growing population of people with cognitive impairment and the lack of effective pharmacotherapeutics, it is prudent for those afflicted or their caregivers to find alternative treatments. Our narrative review shows that many of these nutrients and phytonutrients may be promising for treating some aspects of cognitive impairment, especially for people afflicted with AD.

**Relevance for Patients:**

As demonstrated in a number of clinical trials, healthy adults and patients with various health challenges (e.g., AD, mild cognitive impairment, multiple sclerosis, and Parkinson’s disease) exhibiting a wide range of severity in cognitive defects would be best served to consider multiple nutrients and phytonutrients to improve aspects of their cognitive function.

## 1. Introduction

While cardiovascular disease and cancer continue to be the leading causes of death worldwide [1], shifting trends in morbidity and mortality have resulted in an increasing interest in brain health, particularly driven by the rising incidence of Alzheimer’s disease (AD). Moreover, AD is now the sixth leading cause of death of Americans, and one-third of the elderly will die from AD or another form of dementia [2]. It is currently the only major cause of death and disability that has no efficacious conventional treatment, and it also has no widely accepted preventative strategy. The issue with AD today is reminiscent of that with HIV/AIDS in the early 1990s, before the advent of antiretroviral medication.

Given that the 5 Food and Drug Administration (FDA)-approved drugs for dementia can only delay the decline of AD for a short period of time before continued decline in functioning until death [2], those affected by this disease (e.g., patients and their primary caregivers) have sought alternatives. Aside from strategies such as hyperbaric oxygen treatment, cognitive training, and acupuncture, among others, nutritional science is an evolving field in its application to brain health, in both preventative and restorative study models. In particular, dietary supplements offer an approach to adding key nutrients or phytonutrients to a person’s daily consumption of food and drink. Historically, supplements such as *Ginkgo biloba* and vitamins B12 and E have been studied for their effects on brain health and cognitive functioning among healthy older adults and in those with varying degrees of cognitive impairment. Recently, other nutrients and phytonutrients, such as polysaccharides, saffron, choline, and vitamin D, have also been evaluated for their efficacy on cognitive functioning. This review will summarize the recent findings for these nutrients and phytonutrients and assess whether they offer potential for helping those affected by AD and other neurodegenerative disorders and for those interested in preventing cognitive dysfunction and maintaining brain health. Given the recent rapid increase in the incidence of AD and related diseases, this summary should be of interest to lay people (patients and their caregivers), clinicians, and researchers alike.

## 2. Methods

A comprehensive search for articles was performed using PubMed. Articles published in English between 2000 and 2021 with full text available were searched using the name of the nutrient or phytonutrient and the term “cognitive function/ing.” Inclusion criteria were: (1) a study published within the past 21 years and (2) a clinical trial conducted in humans. Six independent reviewers evaluated the articles for inclusion in the review. The search returned 2,234 total articles, of which 96 were included in this review. See Table 1 for a summary of the nutrient or phytonutrient according to the treatment amount, population under study, and the most significant effects on cognitive function.

**Table 1:** Summary of effects of nutrients and phytonutrients on cognitive function in clinical trials

Nutrient or phytonutrient	Study authors	Study population	Sample size (n)	Daily dose	Main cognitive results
**Antioxidant nutrients**					
α-lipoic acid	Hager *et al*., 2001 [3]	Probable AD and related dementias	Treatment: 9	600 mg/day of α-lipoic acid over an average of 337 days	Cognitive function stabilized according to the MMSE and ADAS-cog
	Hager *et al.,* 2007 [4]	Mild-to-moderate dementia	Treatment: 43	600 mg/day of α-lipoic acid for up to 48 months	Loss of cognitive function was progressively slower in those with mild dementia compared to those with moderate-early and moderate-advanced dementia according to the MMSE and the ADAS-cog
	Shinto *et al*., 2014 [5]	Probable AD	Ω-3: 13 Ω-3 + LA: 13 Placebo: 13	α-lipoic acid/Ω-3 combination 600 mg 3 times/day for 12 months	Less cognitive decline compared to placebo measured with the IADL and when compared to placebo and Ω-3 only measured with the MMSE
Vitamin E (α- and γ-tocopherols)	Dysken *et al.*, 2014 [6]	Mild-to-moderate AD	α-tocopherol: 140 Memantine: 142 Combination: 139 Placebo: 140	2,000 IU/day of vitamin E for average of 2.27 years	Delay of 19%/year in clinical progression (ADL) of AD and attenuated an increase in caregiver time compared to placebo
	Lloret *et al.*, 2009 [7]	Mild-to-severe AD	Treatment: 19 Placebo: 14	800 IU/day of vitamin E for 6 months	Vitamin E respondents had lower blood-oxidized glutathione and scores on cognitive tests (the MMSE, Blessed-Dementia Scale, and Clock Drawing Test) were maintained, whereas when vitamin E was not effective in preventing oxidative stress (non-respondents) cognition decreased to levels lower than those subjects on placebo
N-acetyl cysteine	Breier *et al.*, 2018 [8]	Early schizophrenia	Treatment: 30 Placebo: 30	Escalating doses of NAC (600 mg to 3,600 mg/day) for 52 weeks	Significant decrease in negative symptoms (Positive and Negative Syndrome Scale score), negative symptom factor, and disorganized thought factor compared to placebo, but no improvement in positive symptoms (Positive and Negative Syndrome Scale score)
	Rapado-Castro *et al*., 2017 [9]	Psychosis	Treatment: 27 Placebo: 31	2,000 mg/day of NAC for 24 weeks	Significant improvement in working memory performance as compared to placebo
	Hoffer *et al.*, 2013 [10]	Mild TBI (blast induced)	Treatment before 24 h NAC: 29 Placebo before 24 h: 31 Treatment after (26 – 72 h) NAC: 12 Placebo after (26 – 72 h): 9	4 g loading dose, then 4 g/day for first 4 days, and 3 g/day for the next 4 days of NAC	Significant symptom improvement including resolution of balance dysfunction, headache, confusion, and restoration of TMT performance scores to the age-based norms compared to placebo, and significantly more improvement noted in those who were treated earlier than later with NAC
**Not primarily antioxidant nutrients**					
B vitamins	Aisen *et al*., 2008 [11]	Mild-to-moderate AD	Treatment: 240 Placebo: 169	5 mg folic acid, 1 mg B12 (cyanocobalamin), and 25 mg B6 (pyridoxine hydrochloride) per day for 18 months	No significant change was noted in cognition according to the ADAS-cog
	Durga *et al*., 2007 [12]	Middle-aged subjects with elevated plasma homocysteine	Treatment: 406 Placebo: 413	800 µg/day of folic acid for 3 years	Memory, information processing speed, and sensorimotor speed all significantly improved in the folic acid group compared to placebo
	McMahon *et al*., 2006 [13]	Elderly subjects with plasma homocysteine concentrations ≥13 μmol/liter	Treatment: 138 Placebo: 138	1,000 μg folic acid, 500 μg B12, and 10 mg B6 per day for 2 years	No assessments of cognitive functioning improved
	De Jager *et al*., 2012 [14]	Elderly subjects with MCI	Treatment: 133 Placebo: 133	0.8 mg folic acid, 0.5 mg B12, and 20 mg B6 per day for 2 years	Executive function was maintained in the B vitamin group compared to placebo and in those with baseline homocysteine above the median value (11.3 μmol/L), global cognition (MMSE), episodic memory (Hopkins Verbal Learning Test–delayed recall), and semantic memory (Category Fluency) improved in the B vitamin group compared to placebo
Cholinergic precursors - choline (citicoline), lecithin (phosphatidylcholine), and phosphatidylserine	Cotroneo *et al.*, 2003 [15]	Elderly subjects with mild vascular cognitive impairment	Treatment: 265 Control: 84	1,000 mg/day of citicoline for 9 months	Cognitive function (MMSE) maintained significantly more in citicoline group than control group
	Alvarez-Sabin *et al*., 2013 [16]	Subjects with first-ever ischemic stroke	Treatment: 172 Control: 175	1 g/day of citicoline for 12 months	Attention, executive function, and temporal orientation improved in the citicoline group compared to the control group
	Knott *et al*., 2014 [17]	Healthy subjects with EEG similarities to Schizophrenia	24 (crossover)	Subjects received 4 capsules of either 500 mg or 1,000 mg citicoline in 3 separate testing sessions	Executive function as measured by Groton Maze Learning Task was higher on the 500 mg dose, while auditory gating executive function as indexed by suppression of the P50 event-related potential in a paired-stimulus paradigm was higher on the 1,000 mg dose
	De Jesus Moreno *et al*., 2003 [18]	Mild-to-moderate AD	Treatment: 132 Placebo: 129	400 mg of choline alfoscerate 3 times/day for 180 days	Cognitive function significantly improved as measured by the ADAS-cog
	Kato-Kataoka *et al.*, 2010 [19]	Subjects with mild memory impairment	PS100: 26 PS300: 26 Placebo: 26	100 mg/day or 300 mg/day of phosphatidylserine for 6 months	Significant improvement in delayed verbal recall noted in both phosphatidylserine groups as compared to placebo
	Richter *et al.*, 2013 [20]	Older subjects with age-associated memory impairment	Treatment: 30	300 mg/day of phosphatidylserine for 12 weeks	Significant improvements noted in memory recognition, memory recall, executive functioning, and mental flexibility on the computerized test and total learning and immediate recall on the AVLT
	More *et al.*, 2014 [21]	Elderly subjects with memory problems	Treatment: 40 Placebo: 32	300 mg/day of phosphatidylserine and 240 mg/day phosphatidic acid for 3 months	Significant improvements noted in memory according to the WMS and mood according to the List of Depressive Symptoms as compared to placebo
		AD	Treatment: 55 Placebo: 39	300 mg/day of phosphatidylserine and 240 mg/day phosphatidic acid for 2 months	Daily functioning (i.e., 7 ADLs) maintained in the treatment group significantly more than placebo
Vitamin D	Darwish *et al*., 2017 [22]	Relapse-remitting MS	Deficient/treatment: 41 Sufficient/usual care: 47	Subjects who were 25(OH)D deficient consumed 10,000 IU/day of vitamin D3 for 3 months	Vitamin D3 significantly improved cognitive performance in the 25(OH)D deficient group as compared to 25(OH)D sufficient group, according to the MoCA and the Brief Visuospatial Memory Test
	Hu *et al*., 2018 [23]	Elderly subjects with MCI	Treatment: 93 Placebo: 88	400 IU/day of vitamin D3 for 12 months	Significant improvement noted in several domains of the WAIS-R, e.g., information, digit span, vocabulary, block design, and picture arrangement test, as compared to placebo, and significant improvement noted in full IQ, verbal IQ, and performance IQ in the vitamin D group
	Jia *et al*., 2019 [24]	AD	Treatment: 105 Placebo: 105	800 IU/day of vitamin D for 12 months	Significant improvements noted in several domains of cognitive function, that is, full scale IQ, information, digit span, vocabulary, block design, and picture arrangement, as compared to placebo
	Dean *et al*., 2011 [25]	Healthy adults	Treatment: 63 Placebo: 65	5,000 IU/day of cholecalciferol for 6 months	No significant improvements noted in working memory (N-Back task), response inhibition (Stop-signal task), and cognitive flexibility (Set shifting task)
	Pettersen *et al*., 2017 [26]	Healthy adults with baseline 25(OH)D≤100 nmol/L	Low dose: 40 High dose: 42	Either high dose (4,000 IU/day) or low dose (400 ID/day) vitamin D3 (cholecalciferol) for 18 weeks	Significant improvement noted in nonverbal (visuospatial) memory in high dose group
Omega-3 fatty acids	Chiu *et al*., 2008 [27]	AD and patients with minimum cognitive impairment	Treatment: 24 Placebo: 22	1.8 g/day consisting of 720 mg DHA and 1,080 mg EPA for 24 weeks	Significant improvement in cognition in patients with minimum cognitive impairment, but not in AD patients, and a higher proportion of EPA in red blood cell membranes was associated with better cognitive outcomes
	Shinto *et al*., 2014 [5]	Probable AD	Ω-3: 13 Ω-3 + LA: 13 Placebo: 13	3 g/day consisting of 675 mg DHA and 975 mg EPA and/or 600 mg/day of racemic LA for 12 months	Significantly lesser decline noted in cognitive ability (MMSE) and functional ability (IADL) in the combination group (Ω-3 and LA) versus placebo or Ω-3 alone, and Ω-3 only group also showed lesser decline in functional ability (IADL) as compared to placebo
Zinc	Maylor *et al*., 2006 [28]	Healthy younger and older adults	Younger 0 mg/day: 63 Younger 15 mg/day: 60 Younger 30 mg/day: 65 Older 0 mg/day: 67 Older 15 mg/day: 66 Older 30 mg/day: 66	15 or 30 mg/day of zinc for 6 months	Significant improvements in both zinc groups compared to placebo in cognitive function as measured with spatial working memory errors and matching to sample visual search latency
**Phytonutrients**					
Aloe polysaccharides	Wang *et al*., 2004 [29]	Healthy male college students	Treatment: 10 Placebo: 10	1-time polysaccharide mixture of 1 tablespoon containing 3.9 g of carbohydrates, 0.28 g of protein, and 14 calories	Significant enhancements noted in the power of EEG brain frequencies (theta, alpha, and beta) that were related to attention and arousal in 30 min after consumption as compared to placebo
	Stancil *et al.*, 2009 [30]	College students	62 (crossover)	1-time polysaccharide mixture of 1 tablespoon of Ambrotose Complex in 4 ounces of noncaloric fruit-flavored water	Performance enhancement noted on the visual discrimination task and on the first part of the simple working memory test after consumption
	Best *et al*., 2008 [31]	Middle-age healthy subjects	Polysaccharides: 15 Glucose: 15 Placebo: 15	1-time consumption of either polysaccharides combination (7 g of powdered Ambrotose Complex) or glucose (25 g of powder)	No significant difference in cognitive function was noted, but the scores were higher on immediate and delayed recall and recognition 15 min after consumption compared to glucose
	Best *et al*., 2015 [32]	Middle-age healthy adults	Polysaccharides: 23 Sucrose: 24 Placebo: 26	1-time consumption of either 4 g (1 tablespoon) of a polysaccharide mixture (Ambrotose complex) or 4 g of sucrose (icing sugar)	Significantly higher scores on recognition and working memory 30 min after consumption as compared to the placebo and sucrose groups
	Lewis *et al.*, 2013 [33]	Moderate-to-severe AD	Treatment: 34	Aloe polymannose multinutrient complex formula (20 g/day) for 12 months	Clinically and statistically significant improvements noted in cognitive function (ADAS-cog) at 9 and 12 months follow-up compared to baseline
	McDaniel *et al*., 2019 [34]	Relapse-remitting MS	Treatment: 15	Multinutrient, polysaccharide dietary supplement regimen (~18 g/day) for 12 months	Significant improvements noted in all cognitive functioning and mood symptoms as measured by the Functional Assessment of MS and the Self-Assessment of Severity of MS Symptoms Scale
*Bacopa monnieri* (L.) Wettst.	Sadhu *et al.,* 2014 [35]	AD and healthy elderly individuals	Healthy: 109 AD: 123	Polyherbal formula including *Bacopa monnieri*, sea buckthorn, and dioscorea (500 mg/day) for 12 months	Cognition improved in AD patients (per digit symbol substitution, word recall immediate, and attention span) and in healthy participants (per MMSE, digit symbol substitution, and word recall delayed) as compared to placebo
	Goswami *et al*., 2011 [36]	Newly diagnosed AD	Treatment: 50	300 mg *Bacopa monnieri* standardized extract twice/day for 6 months	Significant improvements in cognition were noted as compared to baseline for orientation of time/place/person, attention, reading, writing, and comprehension measured with the MMSE
	Kumar et al., 2016 [37]	Healthy medical students	Treatment: 28 Placebo: 18	150 mg standardized extract *Bacopa monnieri* twice/day for 6 weeks	Significant improvements noted in attention, freedom from distractibility, and working memory (digit span backwards test), immediate recall of logical material and language comprehension (logical memory test) as compared to placebo
	Calabrese *et al*., 2008 [38]	Elderly subjects without signs of dementia	Treatment: 27 Placebo: 27	300 mg/day of standardized *Bacopa monnieri* extract for 12 weeks	Improvements noted in AVLT (delayed recall) and Stroop Test scores as compared to baseline and decreased anxiety (State-Trait Anxiety Inventory) and improved mood on the Center for Epidemiologic Studies Depression scale
	Stough *et al.*, 2001 [39]	Healthy adults	Treatment: 23 Placebo: 23	300 mg/day of *Bacopa monnieri* extract for 2 weeks	Significantly improved speed of visual information processing (AVLT) and decreased anxiety (State-Trait Anxiety Inventory)
	Stough *et al.,* 2008 [40]	Healthy adults	Treatment: 33 Placebo: 29	300 mg/day of *Bacopa monnieri* extract for 90 days	Significant improvements noted in spatial working memory accuracy and RVIP (Cognitive Drug Research computerized assessment battery) compared to placebo
	Morgan *et al*., 2010 [41]	Elderly subjects	Treatment: 49 Placebo: 49	300 mg/day of *Bacopa monnieri* extract for 12 weeks	Significant improvements noted in verbal learning, memory acquisition, and delayed recall according to AVLT compared to placebo
*Ginkgo biloba* leaf and *Ginkgo biloba* extract (EGb 761)	Attia *et al.*, 2012 [42]	Brain tumor survivors	Treatment: 34	120 mg/day of *Ginkgo biloba* leaf for 24 weeks	Significant improvements noted in executive function (TMT B), attention/concentration (TMT A) and intermediate, delayed recall non-verbal memory (ROCFT), quality of life (Functional Assessment of Cancer Therapy brain and physical subscales), and distressed mood (Profile of Mood States)
	Lewis *et al.*, 2014 [43]	Healthy older subjects with no cognitive deficits	Ginkgo Synergy + choline: 33 OPC Synergy + Catalyn: 31 Placebo: 33	Ginkgo Synergy (120 mg/day *Ginkgo biloba* leaf, 80 mg/day *Ginkgo biloba* whole extract, and other compounds) plus 700 mg/day of choline or OPC Synergy plus Catalyn for 6 months	Significant improvements in time on the TMT B and COWA Trial-S as compared to baseline for the Ginkgo Synergy plus choline group
	Solomon *et al.*, 2002 [44]	Community-dwelling healthy older subjects	Treatment: 115 Placebo: 115	40 mg/day of *Ginkgo biloba* for 6 weeks	No improvements in learning, memory, attention, or concentration
	Mix *et al.*, 2000 [45]	Adults without cognitive dysfunction	Treatment: 24 Placebo: 24	180 mg/day EGb 761 for 6 weeks	Significant improvement noted in speed of processing abilities (Stroop Test) as compared to placebo
	Mix *et al.*, 2002 [46]	Community-dwelling older subjects	Treatment: 131 Placebo: 131	60 mg EGb 761 3 times/day for 6 weeks	Significant improvements noted in delayed free recall and recognition (SRT and WMS-III Faces II) as compared to placebo
	Kaschel *et al*., 2011 [47]	Healthy middle-aged subjects	Treatment: 94 Placebo: 94	240 mg/day of EGb 761 for 6 weeks	Significant improvements noted in the number of correctly recalled appointments (immediate and delayed recall) and the quality of immediate and delayed recall (ratio of false-to-correct) as compared to placebo
	Snitz *et al.*, 2009 [48]	Older subjects with normal cognitive function or minimal cognitive impairment	Treatment: 1,545 Placebo: 1,524	120 mg EGb 761 2 times/day for a median of 6.1 years	No significant improvement was noted in cognitive function as compared to placebo
	Kanowski *et al*., 2003 [49]	Pre-senile and senile AD and multi-infarct dementia	Treatment: 106 Placebo: 99	240 mg/day of EGb 761 for 24 weeks	Statistically significant, but not clinically significant, improvements in cognitive function (SKT and ADAS-cog) compared to placebo
	McCarney *et al*., 2008 [50]	Mild-to-moderate dementia	Treatment: 88 Placebo: 88	120 mg/day of EGb 761 for 6 months	No significant improvement was noted in cognitive function
	Mazza *et al.*, 2006 [51]	Mild-to-moderate AD	*Ginkgo*: 25 Donepezil: 25 Placebo: 26	160 mg/day of EGb 761 for 24 weeks	Significant improvements noted in cognitive function (SKT) and in overall patient condition and therapeutic efficacy (CGI) in EGb 761 and donepezil groups compared to placebo
	Napryeyenko *et al*., 2010 [52]	Probable/possible AD or vascular dementia	Treatment AD: 106 Treatment vascular: 94 Placebo AD: 112 Placebo vascular: 88	240 mg/day of EGb 761 for 22 weeks	Significant improvements noted as compared to placebo in all cognitive function testes used, that is, Short Syndrome Test total scores, Neuropsychiatric Inventory, the Verbal Fluency Test, the Clock-Drawing Test, the Hamilton Rating Scale for Depression, and the Gottfries-Bråne-Steen Scale, and no differences between AD and vascular dementia
	Herrschaft *et al*., 2012 [53]	Mild-to-moderate AD or vascular dementia	Treatment: 205 Placebo: 205	240 mg/day of EGb 761 for 24 weeks	Significant improvements compared to placebo in cognitive function measured with the SKT, Neuropsychiatric Inventory, ADCS CGI of Change, the Verbal Fluency Test, the ADL International Scale overall mean score, the Dementia Quality of Life Instrument-Proxy total score, and the 11-point box scale for dizziness
	Lovera *et al*., 2012 [54]	MS	Treatment: 61 Placebo: 60	240 mg/day of EGb 761 for 12 weeks	No improvement noted in cognitive function as compared to baseline or placebo
	Gavrilova *et al.*, 2014 [55]	MCI	Treatment: 80 Placebo: 80	240 mg/day of EGb 761 for 24 weeks	Improvements noted in cognition and global ratings (TMT A and B) and anxiety (State-Trait Anxiety Inventory), while a trend was noted for the Geriatric Depression scale score
	Beck *et al*., 2016 [56]	Elderly subjects with subjective memory impairment	Treatment: 31 Placebo: 30	240 mg/day of EGb 761 for 60 days	Significant improvement in cognitive flexibility (task-set switch costs) compared to placebo and a trend of improvement in response inhibition (Go-NoGo-task reaction times) compared to placebo
	Li *et al*., 2019 [57]	Vascular MCI patients	Treatment (Pushen): 30 Control (*Ginkgo*): 32	Either 19.2 mg/day of EGb 761 or 1.8 mg 3 times/day of Pushen for 12 weeks	Significant improvements noted for both groups (*Ginkgo* and Pushen) in cognitive function as measured by the MoCA, but only the Pushen group showed significant improvement in the MMSE, while the *Ginkgo* group showed only modest improvement, both groups had higher scores for Subjective Memory Loss, and the Pushen group showed improvement in forgetting acquaintance’s name, while the *Ginkgo* group performed significantly worse for this component as compared to Pushen
Ginseng - *Panax ginseng C.A. Mey* and *Panax* (American) *quinquefolius*	Wesnes *et al.*, 2000 [58]	Middle age healthy subjects	Total: 279	Either of 2 doses of a *Ginkgo biloba*/*Panax ginseng* combination; 160 mg twice daily and 320 mg in the morning daily for 14 weeks	Quality of memory index significantly improved with the *Ginkgo biloba*/*Panax ginseng* combination for both doses, when compared to baseline, as well as with the placebo, but other aspects of cognition such as speed of memory index, continuity of attention, and power of attention did not improve significantly
	Park *et al*., 2019 [59]	MCI	Treatment: 45 Placebo: 45	3 g/day of *Panax ginseng* powder in capsules for 6 months	Immediate recall and delayed recall (measured with the ROCFT) significantly improved in the ginseng group compared to placebo, but no significant improvements were noted in the K-MMSE, K-IADL, and the Seoul Neuropsychological Screening Battery
	Heo *et al.*, 2008 [60]	AD	Low dose: 15 High dose: 15 Control: 31	Either low dose (4.5 g/day) or high dose (9 g/day) Korean red ginseng for 12 weeks	Cognitive and functional performance (measured by ADAS and CDR) significantly improved in the high dose ginseng group versus control
	Heo *et al*., 2012 [61]	Moderately-severe AD	Low dose: 10 Intermediate dose: 10 High dose: 10 Control: 10	Low dose (1.5 g/day), intermediate dose (3 g/day), or high dose (4.5 g/day) of ginseng (SG-135) for 24 weeks	Cognitive function (measured with ADAS-cog and MMSE) significantly improved in the high-dose ginseng group compared to baseline, but no improvements were noted in the low- or intermediate-dose ginseng groups or placebo
	Mariage *et al*., 2020 [62]	Healthy subjects with significant mental workload during workdays	HRG80 (red ginseng): 17 PGS (white ginseng): 16 Placebo: 17	Either 2 capsules/day (418 mg each) of red *Panax ginseng* Meyer root preparation or 2 capsules/day (384 mg each) of white *Panax ginseng* standard preparation for 2 weeks	Error rate (d2 test) significantly reduced and attention (d2 test) significantly improved in the red ginseng group vs placebo, but not in the white ginseng group versus placebo, and memory (the computerized memory test) showed significant improvement in both red and white ginseng groups compared to placebo
	Lee *et al*., 2008 [63]	AD	Treatment: 58 Control: 39	Korean white ginseng (*Panax ginseng* powder) 4.5 g/day daily for 12 weeks	Cognitive function (measured by the ADAS-cog and MMSE) significantly improved in the ginseng group vs control group, but improvement became insignificant after discontinuation of ginseng
	Scholey *et al*., 2010 [64]	Healthy subjects	32 (crossover)	Received a 1-time dose of either 100, 200, or 400 mg of *Panax quinquefolius* extract in a capsule in separate treatments with crossovers occurring after 7 days of wash-out	Cognition significantly improved (within 6 h of treatment) from baseline, and each dose of ginseng showed significant improvements for specific aspects of cognition compared to placebo
	Chen *et al*., 2012 [65]	Stable schizophrenia	Treatment: 32 Placebo: 32	1 capsule (100 mg) of ginseng (HT1001) twice/day for 4 weeks	Compared to baseline, visual working memory significantly improved in the ginseng group but not placebo, and verbal working memory did not improve in the ginseng group, but significantly worsened in the placebo group
Lion’s mane mushroom (*Hericium erinaceus*)	Mori *et al*., 2009 [66]	MCI	Treatment: 15 Placebo: 15	4 tablets (250 mg each) containing 96% lion’s mane powder 3 times/day for 16 weeks	Significant improvement in cognitive function according to the HDS-R in the lion’s mane group compared to placebo
	Saitsu *et al*., 2019 [67]	Over 50 years old with intact cognition	Treatment: 16 Placebo: 15	4 capsules (0.8 g each) of lion’s mane/day for 12 weeks	Significant improvement in cognitive function according to the MMSE in the lion’s mane group compared to placebo
Rhodiola rosea (*Rhodiola rosea* L.)	Darbinyan *et al*., 2000 [68]	Healthy young physicians with nonspecific fatigue	Group A (SHR-5 then placebo): 26 Group B (placebo then SHR-5): 30	Daily dose of SHR-5 (170 mg of *Rhodiola rosea* and 4.5 mg of salidroside) for 2 weeks followed by 2 weeks of wash-out then 2 weeks of placebo (order reversed for the other group)	Significant improvement in the total fatigue index (based on results of 5 tests that measured visual and audial perception speed, attention capacity, and short-term memory as indicators of fatigue) after 2 weeks of taking the SHR-5 dose when compared to placebo
	Cropley *et al*., 2015 [69]	Healthy, mildly anxious university students	Treatment: 40 Control: 41	Daily dose of 200 mg of Vitano (main ingredient is Rosalin (WS 1375), a dry extract from the roots of *Rhodiola rosea*) 2 times daily (30 min before breakfast and 30 min before lunch) for 14 days	Significant improvements in self-reported anxiety and stress, significantly lower levels of anger, depression, and confusion, and significantly improved overall mood in the treatment group at 14 days compared to control; no significant differences in sleep, sleepiness, or cognitive performance between the control and treatment groups
	Fintelmann *et al*., 2007 [70]	Adults with physical and cognitive disabilities	Group 1: 60 Group 2: 60	Vigodana (a vitamin and mineral supplement containing a combination of vitamins E, B6, and B12, folate, magnesium, and *Rhodiola rosea* root extract) taken 2 times/day for 12 weeks. Group 1 took 2 capsules/day after breakfast and group 2 took 1 capsule after breakfast and 1 after lunch daily	Significantly improved physical performance noted at 12 weeks compared to baseline (assessed by 4-point rating scale) and significant improvement in cognitive performance and decrease in cognitive impairment symptoms (assessed by digit connection test and 4-point rating scale) noted in both treatment groups compared to baseline; group 1 showed consistently higher improvements in both physical and cognitive performance by week 12 compared to group 2, though both groups showed significantly improved performance compared to baseline
	Aslanyan *et al*., 2010 [71]	Healthy females with chronic stress	Treatment: 20 Placebo: 20	A single dose of 270 mg of ADAPT-232 (containing a standardized combination extract ratio of 2.8:1 for *Rhodiola rosea*, 1.4:1 for *Schisandra chinensis* (Turcz.) Baill., and 10.5:1 for *Eleutherococcus* *senticosus* Maxim)	Significantly improved performance on d2 Test of Attention and the Stroop Test that measured attention, speed, and accuracy of mental performance in treatment group compared to placebo group
Rosemary (*Rosmarinus officinalis* L.)	Pengelly 2012 [72]	Healthy elderly subjects	28 (crossover)	Received in random order one of 4 doses of dried rosemary (750, 1,500, 3,000, or 6,000 mg) or placebo on 5 separate 1-day treatment sessions every week for 5 weeks	Significant improvements noted for the lowest dose in speed of memory measurements, continuity of attention, quality of working memory as compared to placebo and/or baseline within 6 h of consumption, but the higher dose negatively affected performance and subjective feelings of alertness
	Lindheimer *et al*., 2013 [73]	Young adults with low energy	Rosemary: 26 Black pepper: 26 Placebo: 24	1-time consumption of 1.7 g of rosemary	Transient and statistically insignificant decrements were noted in mental fatigue on a visual analog scale and false alarms during the primary cognitive task in 60 and 90 min after consumption
	Moss *et al*., 2018 [74]	Healthy adults	Rosemary water: 40 Plain water: 40	1-time consumption of 250 mL water infused with rosemary	Small to moderate beneficial effects for performance on several tasks: the Corsi blocks mean span length, serial threes and serial sevens correct responses, RVIP correct responses and errors, and immediate and delayed word recall, and a slight negative effect was noted on fatigue on a visual analog scale
	Moss *et al.,* 2003 [75]	Healthy subjects	Rosemary: 48 Lavender: 48 Control: 48	1-time ambient aromatherapy with 4 drops of rosemary essential oil	Significantly higher scores on a secondary memory subfactor (indicating better accuracy during memory-related tasks) and subjective feelings of alertness and contentedness compared to control, but the control group had significantly quicker responses (speed of memory factor and speed of attention factor)
Saffron (*Crocus sativus* L.)	Akhondzadeh *et al*., 2010 [76]	Mild-to-moderate AD	Treatment: 23 Placebo: 23	30 mg/day of saffron for 16 weeks	Significant improvements in cognitive function (ADAS-cog and CDR-SB) compared to the placebo group
	Akhondzadeh *et al*., 2010 [77]	Mild-to-moderate AD	Saffron: 27 Donepezil: 27	30 mg/day of saffron or 10 mg/day of donepezil for 22 weeks	Improvements in cognitive function (ADAS-cog and CDR-SB) were similar in both groups
	Farokhnia *et al*., 2014 [78]	Mild-to-moderate AD	Saffron: 34 Memantine: 34	30 mg/day of saffron or 20 mg/day of memantine for 12 months	Improvements noted in cognitive function (Severe Cognitive Impairment Rating Scale and Functional Assessment Staging) were similar in both groups
	Tsolaki *et al*., 2016 [79]	Amnestic MCI	Treatment: 17 Control: 18	Saffron extract for 12 months	Significant improvement noted in saffron group (MMSE) while control group deteriorated
Tart cherries (*Prunus cerasus* L.)	Kent *et al.,* 2017 [80]	Older adults with mild-to-moderate dementia	Treatment: 24 Control: 25	200 mL/day of cherry juice for 12 weeks	Significant improvements noted in cognitive performance as measured by the category verbal fluency task, AVLT total, AVLT delayed recall, and AVLT 20-min delayed recall tasks
	Caldwell *et al*., 2016 [81]	Older adults with dementia, older adults, and young healthy adults	Young, healthy: 6 Older adults: 5 Older adults with dementia: 5	1-time crossover consumption of anthocyanin-rich cherry juice in one of 2 dose schemes: (1) a single 300 mL dose at 0 h or (2) 100 mL doses at 0, 1, and 2 h	No improvements in acute cognition as measured with AVLT, pattern and letter comparison, or task-switching tests
	Keane *et al*., 2016 [82]	Middle-aged subjects	27 (crossover)	1-time crossover consumption of either 60 mL of tart cherry concentrate or placebo	No improvement in cognitive function
Turmeric (*Curcuma longa* L.)	Cox *et al.*, 2015 [83]	Healthy adults	Treatment: 30 Placebo: 30	400 mg (~80 mg curcumin) of Longvida (a solid lipid curcumin formulation) for 4 weeks	Significant improvements in sustained attention (digit vigilance task), working memory (serial threes subtraction task), alertness, contentedness, mood, and fatigue in comparison to placebo
	Cox *et al*., 2020 [84]	Healthy older adults	Treatment: 46 Placebo: 43	400 mg (~80 mg curcumin) of Longvida (a solid lipid curcumin formulation) for 12 weeks	Significant improvements in working memory performance on the virtual Morris Water Maze and on serial threes and serial sevens as compared to placebo
	Rainey-Smith *et al*., 2016 [85]	Community-dwelling, cognitively-healthy older adults	Treatment: 80 Placebo: 80	500 mg 3 times/day Biocurcumax, a curcumin formulation for 12 months	Although no significant effects were noted, Biocurcumax did delay cognitive decline as compared to placebo
	Small *et al*., 2018 [86]	Middle age and older adults with intact cognition	Treatment: 21 Placebo: 19	Theracurmin (90 mg twice a day highly bioavailable nanoencapsulated curcumin) for 18 months	Significant improvements in SRT Consistent Long-Term Retrieval, SRT Total, Brief Visual Memory Test-Revised, and attention as compared to placebo, and significant decrements of brain Aβ and tau accumulation in amygdala in brain positron emission tomography scans as compared to placebo
Wild yam (*Dioscorea villosa* L.)	Tohda *et al*., 2017 [87]	Healthy adults	Treatment: 18 Placebo: 13	50 mg/day of diosgenin-rich yam extract for 12 weeks	Significant age-dependent improvement noted in cognitive function (Repeatable Battery for the Assessment of Neuropsychological Status) as compared to placebo
*Withania somnifera* (L.) Dunal - Ashwagandha	Choudhary *et al*., 2017 [88]	Adults with MCI	Treatment: 25 Placebo: 25	300 mg of KSM-66 Ashwagandha taken 2 times/day for 8 weeks	Significantly improved performance on immediate and general memory, executive function, attention, and information-processing speed after 8 weeks of ashwagandha compared to placebo; working memory and visuospatial processing were insignificant
	Chengappa *et al*., 2013 [89]	Bipolar disorder	Treatment: 30 Placebo: 30	500 mg/day of *Withania somnifera* extract (Sensoril) for 8 weeks	Significant improvements in performance on Flanker Test (neutral mean response time), Auditory Digit Span (mean digit span backward), and Penn Emotional Acuity Test (mean social cognition response rating) of *Withania somnifera* compared to placebo at 8 weeks
Xanthines (caffeine)	Soar *et al*., 2016 [90]	Regular caffeine users	43 (crossover)	Caffeinated coffee (~50 mg caffeine) or decaffeinated coffee administered at 2 points 1 week apart	Significantly faster mean reaction time on the Stroop Test and performed significantly better on the Jansari Assessment of Executive Functions (average performance, planning, creative thinking, event based prospective memory, time based prospective memory, and action based prospective memory)
	Smith *et al*., 2013 [91]	Healthy adults	Caffeinated: 64 Decaffeinated: 64	1-time consumption of either caffeinated coffee (65 mg caffeine) or decaffeinated coffee	Significantly faster simple reaction times, indicating increases in speed of encoding and response to a novel stimulus, extroverts performed better in the running memory task, and introverts performed worse, and caffeine improved performance in the mental rotation task for those with high anxiety and hindered performance in those with low anxiety
	Haskell-Ramsay *et al.*, 2018 [92]	Regular coffee drinking young adults	72 (crossover)	1-time crossover consumption of either caffeinated coffee (100 mg caffeine), decaffeinated coffee, or coffee-flavored placebo water	Significantly better digit vigilance accuracy and reaction time, compared to the decaffeinated coffee and placebo groups, respectively, significantly faster RVIP reaction time compared to the placebo group, and significantly better mood and mental fatigue ratings noted as compared to placebo
	Higashi *et al*., 2004 [93]	Healthy adults	Treatment: 14	1-time consumption of either caffeinated coffee (180 mg caffeine) or decaffeinated coffee and then crossed over to the other beverage after at least 1 week	On average, the number of answers per session increased in the caffeine group and decreased in the decaffeinated group, likely due to mental fatigue
	Hindmarch *et al*., 2000 [94]	Habitual caffeine drinkers	30 (crossover)	Caffeinated black leaf tea (either 37.5 mg/1 cup or 75 mg/2 cups), caffeinated black coffee (75 mg/1 cup or 150 mg/2 cups) or bottled water at 4 different times of the day	Caffeinated beverages significantly maintained performance throughout the day (critical flicker fusion task), at the same dose of caffeine, tea produced a rapid increase in the critical flicker fusion threshold between 30 and 90 min post-consumption significantly more than coffee, at the same caffeine dose, compared to tea, coffee was associated with significantly faster reaction times between 10 and 90 min post-consumption
	De Bruin *et al*., 2011 [95]	Healthy adults	26 (crossover)	1-time consumption of 2 servings of black tea (50 mg caffeine and 23 mg theanine/serving) over 60 min	Significantly more correct responses on intersensory attention subtasks (auditory and visual) and responded faster (visual) compared to placebo, and significantly more correct responses for task switching and felt significantly more alert, but less calm compared to placebo
		Healthy adults	32 (crossover)	1-time consumption of 3 servings of black tea (30 mg caffeine and 12 mg theanine/serving) over 90 min	A trend for more correct responses noted for visual unisensory subtask (although statistically insignificant), and correct responses for the task switching and feeling of alertness and contentedness were significantly higher than in placebo
	Dietz *et al*., 2017 [96]	Moderate/habitual consumers of caffeine (100-400 mg/day)	23 (crossover)	Matcha tea or a matcha snack bar (each containing 4 g matcha powder with 67 mg theanine and 136 mg caffeine) or placebo tea or placebo bar; treatment repeated for 4 days and each day cognitive function was assessed	Significant improvement noted in tasks measuring basic attention abilities, and psychomotor speed in response to stimuli as compared to placebo, no significant changes in mood noted, and in most cognitive tasks the drink outperformed the snack bar, particularly in tasks measuring the speed of spatial working memory and delayed picture recognition
	Smit *et al*., 2004 [97]	Chocolate consumers	20 (crossover)	1-time consumption of chocolate with cocoa powder or chocolate containing 250 mg theobromine and 19 mg caffeine	Significantly faster simple reaction time response speeds and significantly higher self-reported energetic arousal occurred after administration of the cocoa powder and caffeine, while significantly better performance on the RVIP task and significantly improved hedonic tone occurred after administration of the caffeine compared to placebo
		Chocolate consumers	22 (crossover)	1-time consumption of 60 g portions (12 squares) of chocolate with no methylxanthines (placebo), low methylxanthines (8 mg caffeine + 100 mg theobromine), or high methylxanthines (20 mg caffeine + 250 mg theobromine)	High methylxanthine significantly increased reaction speed compared to zero methylxanthine for the simple reaction time task, and both low and high methylxanthine significantly improved RVIP performance compared to zero methylxanthine, and mood scores for energetic arousal and hedonic tone were not significantly different between the treatment groups
	Sumiyoshi *et al*., 2019 [98]	Japanese undergraduate students	Dark chocolate: 10 White chocolate: 8	Dark chocolate (26.8 mg caffeine and 197.5 mg theobromine/day) or white chocolate (non-detectable methylxanthine) for 30 days	Dark chocolate significantly increased the number of correct answers on the modified Stroop Test after consumption and maintained a marginally higher number of correct responses at 30 days follow-up, as compared to baseline, dark chocolate significantly increased the total performance (digital cancellation test) after consumption and at 30 days follow-up compared to baseline, and white chocolate did not improve any of the above at any stage

## 3. Results

### 3.1. Nutrients

#### 3.1.1. Antioxidant nutrients

3.1.1.1. α-lipoic acid

Lipoic acid is a metabolic co-factor that comes in several different forms and has been suggested as an anti-inflammatory and neuroprotective treatment for AD. Its diverse mechanisms of action for counteracting the pathology of dementia and AD are: (1) activating choline acetyltransferase, which elevates acetylcholine production; (2) increasing glucose uptake, which increases acetyl coenzyme A production and thus elevates acetylcholine; (3) chelating transition metals that inhibit the formation of hydrogen peroxide and hydroxyl radicals, which may counteract aggregation of amyloid-beta (Aβ) peptide, due to an age-dependent reaction with excess brain metal ions; (4) scavenging reactive oxygen species and lipid peroxidation products; and (5) inducing enzymes of glutathione synthesis and other antioxidant enzymes [99]. In addition, lipoic acid’s low molecular weight allows it to be readily absorbed from the diet to cross the blood-brain barrier. Furthermore, lipoic acid and its reduced form, dihydrolipoic acid, are both potent antioxidants that improve intracellular glutathione levels [100]. Thus, lipoic acid may offer potential for managing neurodegenerative conditions that have known inflammatory and oxidant etiologies or relationships.

In perhaps the first controlled trial of α-lipoic acid in probable AD and related dementias, 9 study subjects were given 600 mg/day in addition to the standard treatment of acetylcholinesterase (AChE) inhibitors (AChEI) over an average of 337 days [3]. AChE degrades acetylcholine, a neurotransmitter, particularly in nerves [101], and cognitive dysfunction in AD is related to a pathophysiological decrease of cholinergic neurons [102]. At the end of the treatment period, cognitive function was stabilized according to the Mini-Mental State Examination (MMSE) [103] and AD Assessment Scale-cognitive score (ADAS-cog) [104]. Before study enrollment, the subjects were declining in their cognitive abilities according to these neuropsychological tests, but α-lipoic acid was able to delay further deterioration.

In a follow-up study by the same group of investigators, 43 patients with mild to moderate dementia being treated with AChEIs were given 600 mg/day of α-lipoic acid for up to 48 months [4]. In those subjects with mild dementia (ADAS-cog<15), the disease progressed extremely slowly (ADAS-cog: +1.2 points/year and MMSE: −0.6 points/year), and in those with moderate severity the disease progression was about twice as fast as the mild group. Nonetheless, the rate of progression for the moderate severity group was far lower than for those who were untreated or only taking an AChEI.

Lipoic acid was utilized in a randomized placebo-controlled trial as an addition to intervention with omega-3 (Ω-3) fatty acids in patients with a diagnosis of probable AD [5]. Thirty-nine subjects aged 55 years or older were enrolled in a 12-month intervention and randomized into one of 3 groups: (1) Ω-3 fatty acids of 3 g/day including 675 mg of docosahexaenoic acid (DHA) and 975 mg eicosapentaenoic acid (EPA; *n*=13); (2) the same Ω-3 fatty acids plus racemic lipoic acid 600 mg/day (*n*=13); or (3) placebo of various excipients and oils (*n*=13). Oxidative stress was the primary outcome, and cognitive and functional performance were secondary outcomes. The combined treatment group showed less decline according to the Instrumental Activities of Daily Living (IADL) scale compared to placebo. The combined treatment also showed less of a decline according to the MMSE compared to placebo and Ω-3 fatty acids alone, but not according to the ADAS-cog. Oxidative stress was not improved by either treatment regimen. Thus, while the combination treatment slowed the cognitive and functional decline in probable AD patients over 12 months, despite no changes in the biomarkers, it is uncertain if the effects were due to the synergy between the compounds or lipoic acid alone, since a lipoic acid-only group was not studied.

3.1.1.2. Vitamin E (α- and γ-tocopherols)

Vitamin E (α- and γ-tocopherols) is an essential fat-soluble vitamin and antioxidant. Dietary sources of vitamin E include nuts, seeds, and vegetable oils. As a potent natural antioxidant, vitamin E scavenges free radicals in the cell membrane and protects polyunsaturated fatty acids from lipid peroxidation [105,106]. The central nervous system (CNS) is especially vulnerable to lipid peroxidation due to its high lipid content, so it is possible that the fat solubility of vitamin E may prove advantageous for protecting these lipids [107]. Systemic oxidative stress is a hallmark of AD and is correlated with cognitive function [7]. In relation to AD pathogenesis, it is proposed that Aβ imparts its neurotoxicity through a cascade of free radicals within the neurons that disrupt functioning [106,108]. Vitamin E can block hydrogen peroxide formation and its resultant free radical cytotoxicity [108].

It has been demonstrated that depletion of vitamin E in an AD mouse model reduced Aβ clearance from the brain and the blood and resulted in Aβ accumulation [105]. Therefore, vitamin E may play an important role in Aβ clearance. Lipid peroxidation was also significantly increased and localized where Aβ had accumulated. Subsequent vitamin E supplementation following depletion partially reduced these effects. These results suggest that in individuals who are genotypically predisposed to AD, vitamin E deficiency may pose a risk factor for acceleration of the disease.

In a retrospective study from the Consortium to Establish a Registry for AD database, patients diagnosed with probable AD who had taken at least 5 mg/day of donepezil and 1,000 IU/day of vitamin E had a significantly lesser decline than patients who received no treatment [109]. Although this finding does not establish efficacy for vitamin E in treating or attenuating AD progression, it demonstrates that vitamin E probably does not interfere with the action of donepezil, an FDA-approved AD treatment. In a study, patients with mild-to-moderate AD taking AChEIs who were given vitamin E treatment of 2,000 IU/day for an average of 2.27 years experienced a 19%/year delay in clinical progression measured by the Activities of Daily Living (ADL) scale and an attenuation of increased caregiver time, compared to placebo [6].

In another study, where 33 AD patients were given either 800 IU/day of vitamin E (*n*=19) or placebo (*n*=14) for 6 months, the concept of respondents and non-respondents to vitamin E supplementation was proposed [7]. Those who experienced a reduction in blood glutathione oxidative stress (respondents) with vitamin E supplementation also saw maintenance of cognitive function, according to the MMSE, Blessed-Dementia Scale, and Clock Drawing Test, while those who did not experience a reduction in oxidative stress (non-respondents) actually had a decrease in cognitive function to a level less than that of those taking placebo. Approximately half of the AD patients were non-respondents and did not see a decrease in markers of oxidative stress [7]. The negative outcomes observed in non-respondents possibly occurred through vitamin E acting as a pro-oxidant and imparting deleterious effects. Because vitamin E is lipophilic, it acts on membrane lipids to scavenge radicals. Oxidized vitamin E can be restored to its antioxidant form by passing the radical to a hydrophilic electron acceptor that is free to move within the aqueous cytoplasm [110]. If the oxidized vitamin E is not subsequently reduced, it can act as a pro-oxidant on surrounding membrane lipids [110]. Based on these findings, it may be beneficial to combine the fat-soluble vitamin E treatment with supplementation of a water-soluble antioxidant, such as vitamin C (i.e., ascorbic acid) for superior antioxidant activity.

3.1.1.3. N-acetyl cysteine (NAC)

NAC is a precursor to glutathione and is the rate-limiting substrate for glutathione synthesis. Thus, supplementation with NAC has been found to increase intracellular glutathione in erythrocytes [111], possibly giving it the potential to exert the same effect in the brain. Since multiple neurodegenerative disorders are characterized by glutathione deficits in the CNS and impaired response to oxidative stress, NAC supplementation may be beneficial in these patients and in those with brain injury.

NAC has been investigated in cases of psychotic disorders, such as schizophrenia and psychosis. Schizophrenia is characterized by negative symptoms and cognitive impairment [112], often leading to poor quality of life and functional deficits [113]. Early schizophrenia is often marked by progressive loss of brain mass, with reductions in cortical thickness [114], which is hypothesized to be caused by oxidative stress [115], inflammation [116], and glutamatergic excitotoxicity [117]. Therefore, the antioxidant effects of NAC, such as increases in glutathione and mitigation of pro-inflammatory cytokine levels [118], as well as its ability to regulate glutamatergic function [119], make it a good candidate for treating chronic schizophrenia.

Thus, 60 subjects with early schizophrenia were randomized for 52 weeks to receive either escalating doses of NAC (600 mg – 3,600 mg/day; *n*=30) or placebo (*n*=30), in addition to stable doses of antipsychotic medications [8]. Those who received NAC had significant decreases in the Positive and Negative Syndrome Scale total score, negative symptom factor, and disorganized thought factor compared to placebo. Although brain morphology did not change, the change in total Positive and Negative Syndrome Scale score was significantly associated with baseline right, left, and total mean cortical thickness, baseline right, left, and middle temporal thickness, baseline right, left, and superior parietal thickness, and baseline left caudal middle frontal thickness at 24 weeks and baseline left superior parietal thickness at 52 weeks in the NAC group, with greater thickness being associated with more improvement. Thus, NAC seems to improve multiple cognitive symptoms of schizophrenia but does not appear to improve Positive and Negative Syndrome Scale positive symptoms. In addition, while NAC treatment does not confer changes in brain morphology, it appears that baseline thickness of multiple regions is positively associated with the degree of symptom improvement. Therefore, adjunctive NAC could potentially improve function in patients with schizophrenia, especially those with less severe progressive brain mass loss.

In another study, 58 patients with psychosis were randomized for 24 weeks to receive either 2,000 mg/day of NAC (*n*=27) or placebo (*n*=31) [9]. NAC significantly improved working memory performance, with significantly higher performance at 24 weeks compared to placebo. Therefore, NAC had a significant impact on cognitive functioning in patients with psychosis through improvements in working memory, a crucial prognostic variable that currently has not been improved with pharmaceutical approaches [120]. Overall, NAC appears to be effective in treating symptoms of cognitive impairment in psychotic disorders.

NAC has also been investigated in treating symptoms of acute conditions, such as mild traumatic brain injury (mTBI). Diagnosis of mTBI requires the occurrence of a traumatic event, with a loss or alteration of consciousness, accompanied by at least 1 neurologic or cognitive symptom (most commonly dizziness) [121]. However, not all TBIs can be treated equally; e.g., treatment for blunt head trauma is not necessarily the same as that for blast injury [122].

Therefore, subjects with mTBI from significant blast exposure during military deployment were randomized to receive either NAC or placebo for 7 days (4 g loading dose, followed by 4 g/day for the first 4 days, and 3 g/day for the remaining 4 days) to investigate if the neuroprotective effects of NAC extended to symptoms of mTBI [10]. In addition, the differential outcome effects of early (within 24 h; treatment *n*=29, placebo *n*=31) and delayed (26-72 h; treatment *n*=12, placebo *n*=9) diagnosis and treatment were examined. The NAC group was significantly more likely to achieve symptom resolution after 7 days of treatment. The number of symptoms on day 7 was attributed independently to both the treatment itself and early treatment initiation, with significantly less symptoms in the early treatment NAC group compared to the delayed treatment placebo group. However, the number of symptoms in the delayed treatment NAC group did not differ significantly from either of the placebo groups. The early treatment NAC group had the best odds of complete symptom resolution at day 7. Regression analysis revealed that NAC treatment was significantly better than placebo, and early intervention was significantly better than delayed intervention. More specifically, early NAC treatment had a significant effect on the resolution of balance dysfunction and absence of headache on day 7. Confusion resolution at day 7 was also significantly associated with early treatment time compared to delay. Treatment with NAC also restored normal Trail Making Tests (TMT) A and B performance within 7 days, reaching equivalent scores to age-based norms, while those in the placebo group remained impaired. TMT time was also impacted by the presence of symptoms. Those with symptoms had prolonged times, while those without had shorter times, indicating that these symptoms impacted cognitive function. Day 3 symptoms were significantly predicted by both NAC treatment status and tympanic membrane perforation, which is a common injury in those with blast exposure. However, for the early treatment group, reduction in day 7 symptoms with NAC treatment occurred independently of tympanic membrane status. Therefore, NAC appears to be an effective way to treat symptoms of mTBI from blast exposure and should ideally be administered within 24 h of injury.

#### 3.1.2. Not primarily antioxidant nutrients

3.1.2.1. B vitamins

The B vitamins, B6 (pyridoxine), B9 (folic acid or folate), and B12 (cobalamin), have known relationships with neuronal development, maintenance, and function, and their deficiencies are linked to dementia (cognitive dysfunction) and psychiatric disorders, such as depression, schizophrenia, and bipolar [123]. B vitamins are crucial for methyl group donation reactions during the synthesis of proteins, lipids, nucleic acids, neurotransmitters, and hormones, e.g., methylenetetrahydrofolate reductase, and B9 and B12 are a part of the methionine synthase complex that reduces homocysteine to methionine [123]. Folate is required for cellular synthesis, repair, and methylation, and it is necessary to keep homocysteine at a normal value and for its methylation to methionine [124]. It has previously been found that serum homocysteine is higher and folate and B12 are lower in persons with dementia and AD [124]. Studies have shown that combinations of B vitamins are generally more effective than a single vitamin treatment, but inconsistencies in the findings on cognitive function may be due to polymorphisms in B vitamin-associated biochemical pathways and methodological differences across studies, e.g., different doses, treatment periods, assessments, and baseline vitamin levels [123,125]. In addition, a recent meta-analysis revealed that although plasma homocysteine, a known risk factor for cognitive impairment and dementia, is lowered with B vitamin intake, cognitive functioning is generally not improved by the same treatment in both adults with and without existing cognitive dysfunction [126]. The lack of benefit on cognitive functioning through B vitamin supplementation could be due to variation in the type of treatment, duration of the intervention, and other study design differences. Nonetheless, some of these findings are discussed below.

In a large multi-center trial executed by the Alzheimer Disease Cooperative Study consortium, 409 subjects with mild-moderate AD (MMSE between 14 and 26) and normal levels of folic acid, B12, and homocysteine were randomized to either (1) 5 mg/day folic acid, 1 mg/day B12 (cyanocobalamin), and 25 mg/day B6 (pyridoxine hydrochloride; *n*=240) or (2) an identical placebo tablet (*n*=169) for 18 months [11]. The ADAS-cog score was the primary outcome, along with other measures of cognition and quality of life, and homocysteine and other biomarkers were secondary outcomes of interest. The change in the ADAS-cog score over the 18-month period was 0.40 points/month for the B vitamin treatment group compared to 0.37 points/month for the placebo group, which was non-significantly different. In addition, changes in all secondary cognition and quality of life measures were also similar, despite a significant reduction in plasma homocysteine in the B vitamin treatment group. Thus, vitamin B supplementation was not able to delay cognitive decline in mild to moderate AD patients.

In another large multi-center study as part of the Folic Acid and Carotid Intima-media Thickness trial, the effect of 3 years of folic acid supplementation was examined on cognitive function in adults 50 – 70 years of age with intact cognitive function, elevated plasma homocysteine, and normal serum vitamin B12 levels [12]. Participants (*n*=819) were randomly assigned to receive 800 μg/day of folic acid (*n*=406) or identical placebo (*n*=413) and were assessed at baseline and 3 years on various measures of cognitive functioning. Serum folate increased and plasma homocysteine decreased in those on folic acid compared to placebo. Changes in memory, information processing speed, and sensorimotor speed all significantly improved in the folic acid group compared to placebo. Thus, folic acid demonstrated improvements in certain domains of cognitive functioning and a decrease in homocysteine, which may prove important for maintaining intact cognition in older normal adults.

Another large clinical trial was conducted in 276 healthy adults aged ≥65 years of age with plasma homocysteine concentrations of ≥13 μmol/liter [13]. Subjects were randomized for 2 years to receive either a daily dose of folate of 1,000 μg, B12 of 500 μg, and B6 of 10 mg (*n*=138) or an identical placebo (*n*=138) and were assessed using a neuropsychological battery of multiple assessments of cognitive functioning at baseline and after 1 and 2 years of intervention. Although plasma homocysteine was significantly lower at 12 and 24 months in the active treatment group compared to placebo, none of the assessments of cognitive functioning differed between groups. Thus, this treatment regimen of B vitamins did not improve cognitive function, despite a reduction in homocysteine.

In a clinical trial of adults aged ≥70 years of age with mild cognitive impairment (MCI), 166 subjects were randomized for 2 years to receive a daily treatment of 0.8 mg of folic acid, 0.5 mg of B12, and 20 mg of B6 (*n*=133) or an identical placebo (*n*=133) [14]. MCI leads to dementia in about 50% of cases, so a remedy to slow or reverse this condition is a public health priority [127]. While the rate of brain atrophy was the primary outcome of interest in the original study, a large battery of cognitive functioning and plasma homocysteine was collected as secondary outcomes. Plasma homocysteine decreased by 30% in the B vitamin group compared to placebo. In addition, executive function, a measure of cognitive function, was maintained in the B vitamin group compared to placebo. In those subjects with baseline homocysteine above the median value (11.3 μmol/L), global cognition (according to the MMSE), episodic memory (according to the Hopkins Verbal Learning Test-delayed recall), and semantic memory (Category Fluency) improved in the B vitamin group compared to placebo. Thus, B vitamin treatment may diminish the rate of cognitive decline in persons with MCI, particularly those with a high level of plasma homocysteine.

3.1.2.2. Cholinergic precursors - choline (citicoline), lecithin (phosphatidylcholine), and phosphatidylserine

Choline is an essential nutrient that must be attained from the diet and is the precursor to acetylcholine, a key metabolic mediator of memory within the brain [128,129]. Phosphatidylserine is the primary phospholipid in the brain and is mainly found in the plasma neuronal membrane. It acts as an antioxidant in the brain and increases brain glucose metabolism [20,21]. Thus, decreasing levels of phosphatidylserine are related to memory impairment [21]. Among other pathological features, AD is characterized by a loss of cholinergic function in the neocortex and hippocampus, which was the basis for the use of AChEI and precursors of acetylcholine, such as choline, lecithin, and phosphatidylserine [128]. However, the results of treatment on cognitive functioning with these cholinergic precursors have been inconsistent at best.

3.1.2.2.1. Citicoline

Citicoline has been hypothesized as a superior form of choline, due to its choline content and its lower susceptibility to being transformed into trimethylamine, a compound that may later undergo hepatic oxidization to trimethylamine N-oxide, a suspected factor in various chronic diseases [130]. Thus, the effects of citicoline on cognitive function have been investigated in various populations. In a study, the efficacy of citicoline was investigated in a large sample (*n*=349) of older people with a mean age of just under 80 years with mild vascular cognitive impairment [15]. The citicoline group (*n*=265) consumed 1,000 mg/day versus no treatment in the comparison group (*n*=84) for 9 months. Change in cognitive function was analyzed from baseline to 3 months and 9 months using the MMSE, and functionality was assessed using the ADL and IADL scales. Overall, significant differences on the MMSE were noted between the citicoline and comparison groups at 3 and 9 months. The ADL and IADL scores were not different between the 2 groups at any time point. Thus, citicoline was effective for improving cognitive function compared to no treatment for patients with mild vascular cognitive impairment, and the compound was well-tolerated without adverse effects.

Cognitive dysfunction is most likely to arise after an initial stroke (compared to subsequent strokes), and stroke also increases the risk of vascular dementia [131]. Thus, treatments are needed to lessen the complications after the first stroke, particularly in maintaining cognitive function. Because citicoline has shown greater efficacy in the early phase of stroke recovery compared to placebo [132], studying its effects on cognitive function after stroke is warranted, particularly in light of the lack of viable treatment options. Thus, in a study of patients with first-ever ischemic stroke, 347 subjects were randomized for 12 months to citicoline (1 g/day) plus usual treatment (*n*=172) compared to usual treatment (*n*=175), 6 weeks after suffering a qualifying stroke [16]. Subjects were assessed on 6 domains of cognitive functioning at 1, 6, and 12 months after stroke. Compared with usual treatment only, patients on citicoline scored higher on assessments of attention-executive function and temporal orientation at 6 and 12 months. Thus, citicoline shows promise in improving some domains of cognitive functioning in the first 12 months of post-stroke recovery and did not cause significant adverse events.

Persons with schizophrenia have lower auditory sensory gating and related cognitive dysfunction [133], likely due in part to altered expression and function of the alpha-7 nicotinic acetylcholine receptor [134,135]. Given that choline is a selective alpha-7 nicotinic acetylcholine receptor agonist, citicoline holds promise as a treatment approach [136]. Thus, healthy adults with electroencephalogram (EEG) similarities to those with schizophrenia (*n*=24) treated with either citicoline or placebo were assessed on executive function using the Groton Maze Learning Task of the CogState Schizophrenia Battery and on auditory gating, as indexed by suppression of the P50 event-related potential in a paired-stimulus paradigm [17]. Subjects were randomized to receive 4 identical-looking capsules of either 500 mg citicoline, 1,000 mg citicoline, or placebo in 3 separate testing sessions, with testing occurring 3 h after administration of the treatment. Subgroup analysis revealed that performance on the Groton Maze Learning Task was better after the 500 mg dose, and gating was better after the 1,000 mg dose. Thus, citicoline showed preliminary benefits for executive function in this sample of subjects with EEG similarities to those with schizophrenia, implying it may be effective in those with the disorder.

3.1.2.2.2. Choline alfoscerate

Mild to moderate AD patients (*n*=261) were randomized to receive choline alfoscerate (400 mg; *n*=132) or placebo (*n*=129) 3 times/day for 180 days to determine its effects on cognitive functioning according to the ADAS-cog, MMSE, and other measures from baseline to after 90 and 180 days of treatment [18]. The ADAS-cog score significantly improved by 2.4 and 3.2 points at 90 and 180 days, respectively, in the choline alfoscerate group, whereas it significantly deteriorated by 0.4 and 2.9 points in the placebo group. Other cognitive measures also generally improved in the choline group compared to placebo. Thus, choline alfoscerate demonstrated significant improvement in cognitive functioning in AD patients without adverse effects.

3.1.2.2.3. Phosphatidylserine

Older Japanese people (50 – 69 years old) with mild memory impairment participated in a randomized trial evaluating the effects of 2 different amounts of soybean-derived phosphatidylserine compared to placebo on various measures of cognition functioning [19]. Eligible subjects (*n*=78) were randomized to take either (1) 100 mg/day of phosphatidylserine (*n*=26), (2) 300 mg/day of phosphatidylserine (*n*=26), or (3) placebo (*n*=26) for 6 months. Hasegawa’s Dementia Scale, Rivermead Behavioral Memory Test, and MMSE, equally improved in all 3 groups at the end of the study, possibly due to practice effects. Nonetheless, those subjects in both phosphatidylserine groups who scored lower at baseline on the cognitive assessments showed significant increases in delayed verbal recall compared to placebo, which did not change. Thus, phosphatidylserine may help some aspects of cognitive functioning among older people who have subjective memory complaints, without adverse effects.

In a smaller study of older adults (aged 50-90; *n*=30) with age-associated memory impairment, soybean-derived phosphatidylserine was evaluated on various measures of cognitive functioning before and after 12 weeks of treatment [20]. Subjects consumed 300 mg/day of phosphatidylserine, and cognitive functioning was assessed with a computerized test battery and the Rey Auditory Verbal Learning Test (AVLT). At the conclusion of the intervention, memory recognition, memory recall, executive functioning, mental flexibility on the computerized test, and total learning and immediate recall on the AVLT all significantly improved. In addition, no adverse effects were noted. Thus, phosphatidylserine may be beneficial for older adults with memory complaints.

A proprietary blend of phosphatidylserine and phosphatidic acid, both made from soy lecithin, was evaluated in 2 groups: (1) non-depressed elderly people with memory problems and (2) patients with AD [21]. In the first group of elderly with memory problems (*n*=72), subjects were randomized for 3 months to 300 mg/day of phosphatidylserine and 240 mg/day phosphatidic acid (*n*=40) or placebo (*n*=32) and were evaluated on memory with the Wechsler Memory Scale (WMS) and mood with the List of Depressive Symptoms. Subjects receiving the phosphatidylserine and phosphatidic acid combination showed significant improvements in memory and feelings of depressed mood compared to the placebo group at the end of treatment. In the second group of patients with AD (*n*=94), participants were randomly assigned for 2 months to either 300 mg/day of phosphatidylserine and 240 mg/day of phosphatidic acid (*n*=55) or placebo (*n*=39) and were assessed on daily functioning, mental health, emotional state, and self-reported general condition. Daily functioning (i.e., performance on 7 ADLs) stayed the same in the treatment group, but declined in the placebo group, and the group difference was significant. Overall deterioration and stability were significantly better in the treatment group compared to placebo. Approximately half of the treatment group reported an overall increase in general condition compared to just over one-quarter of the placebo group. Overall, the combination of phosphatidylserine and phosphatidic acid showed significant improvements in elderly subjects with memory complaints and in AD patients for multiple outcomes, including cognition, mood, daily functioning, and general condition.

3.1.2.3. Vitamin D

Vitamin D is now universally recognized for its importance in preventing a host of chronic diseases, and its deficiency (25-hydroxyvitamin D (25(OH)D) level <20 ng/mL) or insufficiency (25(OH)D level 21 – 29 ng/mL) is a serious global public health challenge, with insufficiency affecting as many as 1 billion people worldwide [137]. Even with the recognized importance of vitamin D for cellular function, the exact insufficiency level is still debated, the desired clinical values are related more to skeletal health, not cognitive outcomes, and the optimal level is also unclear [26,138]. Nonetheless, related to mechanistic aspects of brain function, it has been shown that vitamin D increases acetylcholine levels [139] and hippocampal neuron densities [140] and augments Aβ clearance [141]. In addition, the vitamin D receptor localized in the brain is involved in complex planning, processing, and forming new memories [142], which would portend importance for cognitive function and better overall neurological health [143]. Likewise, it has been shown that both older mildly demented and non-demented adults who were vitamin D deficient were more likely to have mood disorders and performed worse on some assessments of cognitive function [144]. However, reviews of observational, cross-sectional, and case–control studies show that the relationship between vitamin D and cognitive function is inconclusive, owing to methodological issues, disparities between global versus specific assessments of cognitive function, heterogeneity of the populations sampled, and lack of control for confounders [145,146].

Nonetheless, despite the inconsistent associations between vitamin D and cognitive function in observational research, the relationship between vitamin D and cognitive function is significant in patients with multiple sclerosis (MS), who have been shown to be deficient in serum vitamin D [147] and have cognitive dysfunction [148]. Thus, supplementing with vitamin D may hold promise in improving cognitive function among this population. In a study, relapse-remitting MS patients (*n*=88) being treated with IFN beta who had a deficient 25(OH)D level (<25 ng/ml; *n*=41) were compared to MS patients with a sufficient 25(OH)D level (>35 ng/ml; *n*=47) on cognitive performance from baseline to 3 months follow-up [22]. The subjects who were 25(OH)D deficient consumed vitamin D3 10,000 IU/day and those who were 25(OH)D sufficient continued with usual treatment, with the possibility of taking vitamin D3 at varying levels. The cognitive function battery included the Montreal Cognitive Assessment (MoCA), the Stroop Test, the Symbol Digit Modalities, and the Brief Visuospatial Memory Test. At 3 months, vitamin D3 significantly improved cognitive performance in the 25(OH)D deficient group, according to the MoCA and the Brief Visuospatial Memory Test.

As previously mentioned, older adults who are vitamin D deficient may be more prone to cognitive dysfunction. In addition, given the problem of an increasing elderly population with a rising prevalence of MCI [127], the use of vitamin D as a treatment may help to avoid advancing from MCI into dementia. Thus, in a study of elderly 65 years of age or older with MCI (*n*=181), subjects were randomized for 12 months to receive either 400 IU/day of vitamin D3 (*n*=93) or placebo (*n*=88), to determine the effect on cognition and lipid concentrations [23]. Subjects were not able to use dietary supplements known to interfere with nutrition status, vitamin D metabolism, or cognitive function within the 3 months before the trial. Subjects were assessed at baseline and 6 and 12 months follow-up on the MMSE and the Wechsler Adult Intelligence Scale (WAIS)-Revised (WAIS-R), which includes 11 domains of cognitive function. Serum 25-D (the less active precursor) and 1,25-D concentrations were significantly higher in the vitamin D group compared to placebo. Concentrations of lipids (i.e., triglycerides, total cholesterol, high-density lipoprotein cholesterol, and low-density lipoprotein cholesterol) significantly decreased in the vitamin D group and increased in the placebo group. Several domains of the WAIS-R, e.g., information, digit span, vocabulary, block design, and picture arrangement test, significantly increased at the 12-month follow-up in the vitamin D group versus placebo. Full intelligence quotient (IQ), verbal IQ, and performance IQ also significantly increased at the 6- and/or 12-month follow-up assessments in the vitamin D group. Thus, daily vitamin D3 supplementation of 400 IU in a group of elderly with MCI showed promising effects on cognitive functioning, which were hypothesized to at least be partially related to decreases in blood lipids.

AD is a serious public health crisis with no known efficacious conventional therapy for treating the condition, and its effects are costly and widespread from the individual with the disease to society. In addition, vitamin D deficiency is highly related to a greater risk of developing dementia [149]. Thus, it is imperative to evaluate treatments such as vitamin D with known neurocognitive effects in AD that may provide some benefit to these patients, who otherwise are left with an ominous prognosis. In a study, 210 AD patients 65 years of age or older were randomized for 12 months to receive either 800 IU/day of vitamin D (*n*=105) or placebo (*n*=105) to determine the effects on cognitive function and Ab-related biomarkers [24]. Subjects were assessed at baseline and 6 and 12 months with the WAIS-R, ADL scale, the MMSE, and blood draws. The vitamin D group showed significant increases in serum 25-D and 1,25-D at follow-up compared to baseline and placebo. The vitamin D group also scored significantly higher at 12 months and compared to placebo in several domains of cognitive function, e.g., full-scale IQ, information, digit span, vocabulary, block design, and picture arrangement. While both groups’ scores significantly declined for arithmetic, the change was lesser in the vitamin D group. Finally, the vitamin D group significantly improved at 12 months and compared to placebo in several blood Aβ-related biomarkers (i.e., AB42, APP, BACE1, APPmRNA, and BACE1mRNA), providing mechanistic support for the clinical enhancements. Thus, 800 IU/day of vitamin D3 for 12 months improved various domains of cognitive function and several Aβ-related biomarkers in AD patients, showing promise in a disease with few existing treatment options.

Given that 25(OH)D insufficiency or deficiency is common among the general population and in the elderly in particular [145], it is important to study vitamin D treatment among healthy individuals to determine if it may be of benefit for cognitive function. In a study of younger healthy adults, 128 subjects were randomized for 6 weeks to receive either 5,000 IU/day of cholecalciferol (*n*=63) or placebo (*n*=65) to investigate the effects on cognitive functioning and secondary emotional measures [25]. Subjects were assessed on working memory (N-Back task), response inhibition (Stop-signal task), and cognitive flexibility (Set shifting task) at baseline and at 6 weeks. While the group receiving vitamin D significantly increased serum 25(OH)D and the placebo group did not, no measure of cognitive functioning improved at 6 weeks follow-up.

On the other hand, in another clinical trial of healthy adults, 82 subjects with baseline 25(OH)D≤100 nmol/L were randomized for 18 weeks to either high dose (4,000 IU/day; *n*=42) or low dose (400 ID/day; *n*=40) vitamin D3 (cholecalciferol) to evaluate the resultant effect on cognitive function according to the Symbol Digit Modalities Test, verbal (phonemic) fluency, digit span, and the Cambridge Automated Neuropsychological Test Battery (CANTAB) computerized battery [26]. Serum 25(OH)D level increased significantly more in the high dose group. Performance in nonverbal (visuospatial) memory improved in the high dose group (approaching statistical significance after adjusting for demographics and baseline performance), and those with lower baseline 25(OH)D (<75 nmol/L) level in the high dose group improved significantly. The results suggest that an increased 25(OH)D level is crucial for higher executive functioning, such as nonverbal memory. Overall, vitamin D shows preliminary support for its use in improving cognitive functioning in a variety of populations, with a particular emphasis on bettering the lives of those afflicted with AD. Vitamin D supplementation should continue to be evaluated for its efficacy.

3.1.2.4. Ω-3 fatty acids

Ω-3 fatty acids are polyunsaturated fatty acids with anti-inflammatory properties. They inhibit the conversion of highly reactive Ω-6 arachidonic acid into pro-inflammatory factors [150], decreasing T-cell proliferation [150], and inhibiting leukocyte migration [151]. The three Ω-3 essential fatty acids are DHA, EPA, and α-linolenic acid. DHA and EPA are plentiful in fish oil, while α-linolenic acid can be found in plant oils and is especially plentiful in flaxseed [152]. DHA is highly concentrated in the neuronal and synaptic membranes [153], facilitating its anti-inflammatory activity in the brain.

Long-chain Ω-3 has a direct impact on AD pathology by reducing Aβ production, minimizing its aggregation into plaques, and increasing Aβ clearance [154]. Depletion of DHA can result in an increased Ω-6:Ω-3 fatty acid ratio, which creates a physiological environment of inflammation and excessive oxidative stress [155,156]. Both inflammation and oxidative stress are conditions known to enhance the production of Aβ plaques. Brain DHA levels tend to decrease with age, even more so in AD patients [157]. Serum and brain levels of DHA are reported to be lower in AD patients compared to normal adults [158], either due to the low dietary intake of Ω-3 fatty acids or increased oxidative stress.

Several clinical trials have investigated the efficacy of Ω-3 supplementation in adults at risk for AD and cognitive decline. Ω-3 fatty acid consumption yielded a modest attenuation of cognitive decline in the elderly without dementia, but was not associated with prevention or treatment of AD [159]. Mechanisms behind the potential benefits of Ω-3 fatty acids warrant further study into their efficacy for the prevention and treatment of AD. Ω-3 supplementation produced no adverse effects and improved the clinical condition in AD and MCI patients aged 55 – 90 years (*n*=46) [27]. Participants who supplemented Ω-3 (1.8 g/day consisting of 720 mg DHA and 1080 mg EPA for 24 weeks; *n*=24) improved in general health compared to placebo (*n*=22), likely due to cardiovascular and immunological health improvements resulting from Ω-3 supplementation. Patients with MCI on the Ω-3 diet saw a significant improvement in cognition, but this effect was not seen in patients with mild-moderate AD. In addition, a higher proportion of EPA in red blood cell membranes was associated with better cognitive outcomes.

In another study, 39 individuals 55 years and older with a diagnosis of probable AD were given either an Ω-3 supplement (3 g Ω-3/day with 675 mg DHA and 975 mg EPA; *n*=13), a combination Ω-3 and lipoic acid supplement (3 g Ω-3/day plus 600 mg racemic lipoic acid; *n*=13), or placebo (*n*=13) [5]. Oxidative stress did not differ significantly between the treatment and placebo groups. The treatment group that received a combination of Ω-3 and lipoic acid had less of a decline in cognitive function (according to MMSE) and functional ability (measured by IADL) over 12 months. The group that received Ω-3 only also showed less of a decline in IADL compared to the placebo. In both treatment groups, EPA and DHA in the red blood cell membranes significantly increased from baseline. Because these positive effects of treatment were observed without a concurrent decrease in markers of oxidative stress, it is possible that Ω-3 and Ω-3 + lipoic acid may impart their benefits against AD pathogenesis in ways unrelated to oxidative stress.

3.1.2.5. Zinc

Zinc is a key antioxidant for the CNS, influencing brain structure and function [160], especially in the hippocampus and amygdala [161]. Therefore, zinc deficiency can delay physical and cognitive development [162]. Older adults are especially prone to zinc deficiency [163]. A study found that only 44% of adults over 70 years of age had sufficient zinc status [164]. An insufficient zinc level is considered a significant clinical problem in this population [165]. For example, reduced hippocampal zinc has been linked to an age-related decline in spatial memory [161]. However, studies in older adults have found that greater dietary intake of zinc was associated with better cognitive function [166], the cause has not yet been established.

In a study, healthy younger (55 – 70 years; *n*=188) and older (70 – 87 years; *n*=199) adults were randomized for 6 months to receive either 15 or 30 mg/day of zinc or placebo [28]. Cognitive function was assessed at baseline and after 3 and 6 months of supplementation using parallel versions of the CANTAB at each session. At baseline, zinc intake was similar between both groups, but the erythrocyte zinc level was significantly higher in the younger group compared to the older group. Urinary zinc level was significantly lower in the younger group, but serum zinc was not significantly different between groups. Urinary and serum zinc showed dose-dependent increases with supplementation, independent of age. At baseline, serum zinc and pattern recognition memory latency, erythrocyte zinc and pattern recognition memory latency for males and between 5-choice reaction time and erythrocyte zinc for females were significantly correlated. At baseline, younger adults excelled comparatively at cognitive task performance, as they were significantly more accurate (pattern recognition memory, spatial working memory, and spatial span), faster (pattern recognition memory, matching to sample visual search, 5-choice reaction time, and 5-choice movement time), and more efficient (spatial working memory strategy) compared to older adults. Significant interactions between treatment and time were noted for spatial working memory errors (with greater improvements from baseline to 3 months for the 2 treatment groups compared to placebo and with greater improvements from months 3 to 6 for the 15 mg group compared to the 30 mg group) and matching to sample visual search latency (due to greater improvements over 6 months for placebo and 30 mg compared to 15 mg). However, the analysis did not provide any clear evidence that either of the 2 treatment doses provided a substantial or lasting benefit for spatial working memory error compared to placebo. These findings were also independent of sex, education, baseline zinc intake, and serum zinc. Therefore, age-related deficits for performance on the CANTAB were discovered, and the effects of zinc supplementation on cognitive function were limited. The beneficial effect of both doses on spatial working memory errors was only found at 3 months, and the effect of the 15 mg dose on matching to sample visual search latency was detrimental. Thus, further investigation is warranted, perhaps in a more vulnerable and/or zinc deficient population.

### 3.2. Phytonutrients

#### 3.2.1. Aloe polysaccharides

Complex polysaccharides, e.g., arabinose, mannose, xylose, rhamnose, and fucose, are important compounds utilized in the bio-assembly line process by every cell in the body [167]. Most importantly, during the second major step of biosynthesis in all cells, 9 molecules of mannose, a key polysaccharide, are required in the endoplasmic reticulum to initiate the assembly of glycoproteins and glycolipids. This process demonstrates that polysaccharides are not only metabolized to provide energy, but that they are also used for glycosylation (i.e., the addition of saccharides to amino acid chains or free fatty acid chains) in the endoplasmic reticulum and the Golgi. The significance of the coding capacity of polysaccharides in glycoproteins and glycolipids is provided in a series of review articles in a glycomics-dedicated issue of *Acta Anatomica* [168]. The addition of 9 molecules of mannose in 3 chains in the endoplasmic reticulum constitutes the establishment of a domain on which instructions for life processes are transmitted between cells. In the Golgi, other polysaccharides are added, and the number of mannose units is modified to provide a code of information for conducting host defense, repair, growth, healing, and homeostasis. This domain is the principal site for coordinating activities for the trillions of cells that make up the human body. This fundamental supporting biochemistry is why supplying polysaccharides from food sources results in a broad spectrum of health-supporting benefits. In addition, concentrated levels of polysaccharides in dietary supplements, compared to their levels in foods, are advantageous in that a higher amount of nutrients allows a greater number of bioactive compounds to be created. Thus, innate mechanisms of defense and repair coded in the genes can be enhanced to be more effective against infectious agents and compromises in health.

These polysaccharides come from many plants, e.g., rice bran, aloe vera, dioscorea, and others, and they have been characterized by several different investigators [169-171]. Polysaccharides have diverse structure, composition, and molecular heterogeneity that lend them to a wide variety of mechanisms of action, e.g., the effects on colonic microflora and gastrointestinal physiology, immunomodulation, anti-neoplastic, and wound healing, among others [172]. The capacity for polysaccharides to affect neurobiology and resultant cognitive function is not entirely clear and has not been well identified, despite the fact that glucose, a monosaccharide, is the major source of fuel for the brain [29]. Nonetheless, it has been shown that glucose affects memory function directly in the hippocampus [173] and through indirect hormone signaling [174]. Polysaccharides have been shown to combine with proteins and lipids for structural development (i.e., creation of glycoconjugates) of the brain, perform synaptogenesis, and enable the formation of neurotransmitters, all of which ultimately help to determine cognitive function [29,31,175]. In addition, in 20 healthy male college students, compared to placebo (*n*=10), a 1-time polysaccharide mixture (a proprietary blend of 1 tablespoon of all-natural ingredients containing 3.9 g carbohydrates, 0.28 g protein, and 14 calories; *n*=10) 30 min after consumption showed significantly enhanced power according to EEG activity in 3 brain wave frequencies (theta, alpha, and beta) that are related to attention and arousal [29]. Thus, the basis for clinical evaluation of the effects of polysaccharides on cognitive function is justified and warranted.

In an evaluation of the effect of consuming a polysaccharide formula 1 time on cognitive function and memory, 62 college students participated in a study of 2 separate neuropsychological tests [30]. Subjects were randomized to receive a polysaccharide product (1 tablespoon of Ambrotose Complex in 4 ounces of noncaloric fruit-flavored water) or placebo (1 tablespoon of rice starch in noncaloric fruit-flavored water) before testing, and the other drink after testing, or vice versa. In the first test, subjects (*n*=30) completed the Standard Progressive Matrices, the Stroop Test, and the Same-Different visual discrimination task. In the second test, subjects (*n*=32) completed the Reading Span and Operation Span tasks as measures of simple and complex working memory, respectively. Those subjects who consumed the polysaccharide formula before assessment performed more correctly on the visual discrimination task and on the first part of the simple working memory test. Thus, acute consumption of a mixture of polysaccharides may offer an advantage for short-term cognitive performance in certain domains.

In the first of 2 studies conducted by the same research group, middle-aged healthy men and women (*n*=45) were randomized to determine the effect on memory performance of an acute 1-time administration of a combination of either polysaccharides (7 g of powdered Ambrotose Complex; *n*=15), glucose (25 g of powder; *n*=15), or a placebo (2 drops of concentrated liquid stevia; *n*=15), all mixed in a 300 mL low-calorie, and raspberry-flavored drink [31]. Subjects were assessed with a battery of memory tests 15 min after drinking their assigned treatment. The tests consisted of immediate and delayed recall, recognition, short-term memory, working memory, and general cognitive function. While performance on the tests did not differ significantly among the treatments, higher scores on immediate and delayed recall and recognition were noted for the polysaccharide treatment compared to the glucose treatment. Thus, the polysaccharide combination may affect measures of memory performance rather than the performance of working memory.

In the second study, another group of healthy middle-aged adults (*n*=73) participated in an experiment to determine if a polysaccharide mixture was more effective than a rice flour placebo or a sucrose control on measures of mood and cognitive function that was administered to create mental fatigue [32]. Subjects were randomized to a 1-time consumption of either 4 g (1 tablespoon) of a polysaccharide mixture (*n*=23; Ambrotose complex), 4 g of a rice flour placebo (*n*=24), or 4 g of a sucrose (icing sugar) control (*n*=26) in 100 mL of water. Subjects were assessed at baseline, consumed their treatment, waited for 30 min, and then were assessed with different forms of the same tests. The group that took the polysaccharide mixture had significantly higher scores on recognition and working memory compared to the placebo and control treatments, and these differences were unrelated to changes in blood glucose. Thus, the results suggest that certain aspects of cognitive function can be improved with a mixture of polysaccharides that affect metabolic pathways other than increased blood glucose.

While acute cognitive performance studies may help to create a framework to understand how polysaccharides affect neurobiology, the impact of polysaccharides on neurodegenerative disorders like AD is a crucial question to answer, given the current inability of conventional medicine to offer solutions for these patients. As AD has continued to elude an efficacious conventional treatment and the intake of key polysaccharides has been shown to increase the production of adult stem cells [176], a pilot study of 48 AD patients showed that a polysaccharide multinutrient formula had a beneficial effect on overall quality of life [177]. That promising result led to a more formal open-label controlled trial to investigate the effect of an aloe polymannose multinutrient complex formula on cognitive and immune functioning over 12 months among adults diagnosed with moderate to severe AD [33]. Subjects (*n*=34) consumed 4 teaspoons/day (~20 g/day) of the formula and were assessed at baseline and 3, 6, 9, and 12 months follow-up with the ADAS-cog, MMSE, AD Cooperative Study-ADL, and the Severe Impairment Battery. Cytokines and lymphocyte and monocyte subsets were assessed at baseline and 12 months. While the other assessments did not detect changes, the ADAS-cog cognition scores clinically (≥4-point change) and statistically significantly improved at 9 and 12 months from baseline. In addition to the significant finding on the ADAS-cog, multiple immune and inflammatory markers improved at 12 months follow-up. Participants tolerated the dietary supplement with few adverse reactions.

A subsequent analysis on the same sample of AD patients was conducted to determine if the aloe polymannose multinutrient formula resulted in any significant relationships between mature brain-derived neurotrophic factor (BDNF) and its precursor proBDNF and the battery of cognitive functioning measures [178]. As BDNF is key in neurosynaptic function, apoptosis, plasticity, long-term potentiation, learning memory processes, and higher-order thinking [179-184], it could be a useful target for the treatment of AD. While proBDNF and BDNF did not significantly change from baseline to 12 months follow-up, the correlations between the ADAS-cog total score and BDNF and BDNF/proBDNF ratio were statistically significant at 12 months. Other correlations were noted for various cognitive functioning assessments and BDNF and/or BDNF/proBDNF at 12 months, suggesting that the aloe polysaccharide multinutrient formula was consequential on these relationships.

A similar study was executed by the same group on relapse-remitting MS patients (*n*=15) who consumed a multinutrient and polysaccharide dietary supplement regimen (~18 g/day) for 12 months to determine its impact on multiple biomarkers (infections, cytokines, growth factors, and T- and B-cell subsets) and self-report and clinician-administered measures [34]. Cognitive functioning and mood symptoms were components of 2 separate measures, the Functional Assessment of MS and the Self-Assessment of Severity of MS Symptoms Scale, utilized in the battery that was given at baseline and quarterly for 12 months. All of the cognitive functioning and mood symptoms showed statistically significant improvements over the course of the intervention. In addition, at 12 months, total infections decreased significantly, and inflammatory markers and immune functioning significantly improved. Thus, the results of the research on AD and MS patients showed promising effects of polysaccharide formulae on various components of cognitive function in these patient populations that otherwise have few efficacious conventional treatment options.

#### 3.2.2. *Bacopa monnieri* (L.) *Wettst*.

*Bacopa monnieri* is an ancient plant from the Scrophulariaceae family that thrives in damp locations. Both its leaves and stem have medicinal uses. In Ayurveda, the ancient Indian system of medicine, *Bacopa monnieri* has been used for the treatment of neurological disorders associated with intellectual decline and memory loss, such as AD [185].

The main bioactive components of *Bacopa monnieri* are non-polar molecules, bacosides, that can easily cross the blood–brain barrier, leading to anti-inflammatory and antioxidant effects directly within the brain. This is essential in *Bacopa monnieri*’s role against AD, as multiple studies have shown that inflammatory compounds and reactive oxygen species, such as hydroxyl radical and nitric oxide, cause stress-mediated neurodegeneration in this disease [186]. In addition to various measures of cognitive functioning, these anti-inflammatory and antioxidant effects were studied in a clinical trial of 12 months in healthy elderly individuals (*n*=109) and AD patients (*n*=123) [35]. The healthy subjects were randomized to either a polyherbal formula including *Bacopa monnieri*, sea buckthorn, and dioscorea (500 mg/day) or a placebo, and the AD subjects were randomized to either the polyherbal formula or donepezil (20 mg/day). The results indicated that cognitive functioning improved in subjects with AD (according to digit symbol substitution, word recall immediate, and attention span) and healthy subjects (according to the MMSE, digit symbol substitution, and delayed word recall) who were taking the polyherbal formula compared to their respective placebo subjects. The memory-enhancing effects were hypothesized to be due to the polyherbal formula’s ability to decrease markers of both inflammation (i.e., homocysteine, C-reactive protein, and TNFa) and oxidative stress (i.e., glutathione peroxidase, glutathione, and thiobarbituric acid).

In another trial of newly diagnosed AD patients, subjects (*n*=50) between the ages of 60 and 65 years were given 300 mg of *Bacopa monnieri* standardized extract twice/day for 6 months [36]. Subjects were compared on the MMSE from baseline to 6 months, and statistically significant improvements were noted for orientation of time/place/person, attention, reading, writing, and comprehension at the end of the trial. Additionally, self-reported quality of life improved at the conclusion of the study, primarily due to decreased irritability and insomnia.

It is known that the brains of AD patients have a higher level of lipid peroxidation created by oxidative processes [187], which can be decreased by *Bacopa monnieri*, especially in the hippocampus, prefrontal cortex, and striatum [188]. Extracellular Aβ deposits in senile plaques, intracellular neurofibrillary tangles, reactive microgliosis, and astrogliosis are hallmarks of AD, and numerous studies indicate that bacosides protect the brain against oxidative damage and age-related cognitive deterioration by preventing Aβ aggregation and formation of fibrils and by protecting neurons against Ab-induced toxicity [186].

Even though the findings in AD patients have been positive, *Bacopa monnieri* has primarily been evaluated for its effectiveness on cognitive function in healthy adults. In a clinical trial with healthy medical students, subjects (*n*=46) were randomized to either 150 mg of a standardized extract of *Bacopa monnieri* (*n*=28) or placebo (*n*=18) twice daily for 6 weeks and were assessed on a comprehensive assessment of cognitive functioning [37]. At the 6-week assessment, the digit span backwards test (measuring attention, freedom from distractibility, and working memory) and the logical memory test (evaluating immediate recall of logical material and language comprehension) significantly improved in the *Bacopa monnieri* group compared to placebo, but no other tests changed. No adverse effects were reported, showing that treatment with *Bacopa monnieri* was safe.

In a clinical trial of adults age 65 or older without signs of dementia, subjects (*n*=54) were randomized for 12 weeks to receive either 300 mg/day of standardized *Bacopa monnieri* extract (*n*=27) or placebo (*n*=27) to determine its effect on cognition and mood [38]. The primary outcome measure was the delayed recall score from the AVLT, and additional cognitive tests included the Stroop Test, the Divided Attention Task, and the WAIS letter-digit test. Mood was assessed with the State-Trait Anxiety Inventory, Center for Epidemiologic Studies Depression scale, and the Profile of Mood States. The participants who took the *Bacopa monnieri* extract had improved AVLT and Stroop Test scores, whereas the placebo group’s scores were unchanged. No significant differences were found for the Divided Attention Task or the WAIS. The *Bacopa monnieri* group also showed improved State-Trait Anxiety Inventory and Center for Epidemiologic Studies Depression scale scores, and few adverse effects were reported.

In the first of 2 trials of healthy adults from the same research group, subjects (*n*=46) aged 18 to 60 years were randomly assigned for 12 weeks to either 300 mg/day of *Bacopa monnieri* extract (*n*=23) or placebo (*n*=23) [39]. Cognitive function was assessed with a comprehensive battery including the Cognometer tests of working memory, the Digit Symbol Substitution Test, Speed of Comprehension Test, Digit Span, TMT, AVLT, and Inspection Time, and state anxiety was assessed with the State-Trait Anxiety Inventory. The results at 12 weeks showed significantly increased speed of visual information processing according to the Inspection Time task, improved learning rate and memory consolidation on the AVLT, and decreased anxiety in the *Bacopa monnieri* group. In their second trial, healthy participants (*n*=62) aged 18 – 60 years old were randomized for 90 days to either 300 mg/day of *Bacopa monnieri* extract (*n*=33) or a matching placebo (*n*=29) to determine its effects on cognition [40]. Cognitive functioning was assessed with the Cognitive Drug Research computerized assessment battery that included 5 domains (secondary memory, working memory, speed of memory, speed of attention, and accuracy of attention) and a rapid visual information-processing task. Spatial working memory accuracy of the working memory domain and the number of false-positives recorded in the Rapid Visual Information Processing (RVIP) task significantly improved in the *Bacopa monnieri* group compared to placebo at the end of treatment.

In another study, healthy elderly subjects (*n*=98) over 55 years of age were randomized for 12 weeks to receive 300 mg/day of *Bacopa monnieri* extract (*n*=49) or an identical placebo (*n*=49) [41]. The neuropsychological and memory assessment included the AVLT, the Rey-Osterrieth Complex Figure Test (ROCFT), the TMT, and the Memory Complaint Questionnaire. Verbal learning, memory acquisition, and delayed recall according to the AVLT significantly improved in the *Bacopa monnieri* group compared to placebo at 12 weeks, while the other assessments showed minor improvements that did not differ significantly between the 2 groups. The *Bacopa monnieri* group also reported some incidents of gastrointestinal side effects. Thus, several clinical trials show significant and consistent improvements in various domains of cognitive functioning from 3 to 12 months across samples of healthy adults and those with AD with minimal adverse effects, primarily related to gastrointestinal upset.

#### 3.2.3. *Ginkgo biloba* leaf and extract (EGb 761) - *Ginkgo biloba* L.

*Ginkgo biloba* is thought to be beneficial to human health as a neuroprotective agent, antioxidant, free-radical scavenger, membrane stabilizer, and inhibitor of platelet-activating factor (through terpene ginkgolide B). It has also been shown to stimulate choline uptake in the hippocampus and to inhibit Aβ deposition [189]. EGb 761 is the standard preparation of *Ginkgo biloba* extract that contains 24% ginkgo flavonoid glycosides, 6% terpene lactones, and no more than 5 ppm ginkgolic acids [189], and its neuroprotective benefits may be due to its capacity to reduce oxidative damage and stimulate apoptosis [190]. Several studies assessing herbal therapies for the treatment of cognitive deficits have focused on *Ginkgo biloba* and its EGb 761 extract, based on its multifaceted therapeutic potential [45,191-193]. In fact, compared to other nutrients, phytonutrients, and natural compounds, *Ginkgo biloba* and EGb 761 may be the most widely studied material for cognitive functioning in both healthy adults and those with early to late cognitive impairment or dementia. For example, a meta-analysis was conducted among studies of AD patients in 3 – 6 month treatment periods with 120 – 240 mg/day of *Ginkgo biloba* and revealed a small though significant effect size of 0.40 (comparable to the effect of donepezil, which is 0.42-0.48) on objective measures of cognitive function (i.e., ADAS-cog) [194]. According to a summary review of studies at that time, Ernst found that *Ginkgo biloba* was effective at improving memory, concentration, fatigue, anxiety, and depressed mood [195].

3.2.3.1 *Ginkgo biloba* leaf

In a phase II open-label study, symptomatic irradiated brain tumor survivors (*n*=34) were given 120 mg/day of *Ginkgo biloba* for 24 weeks followed by a 6-week washout period to determine its effect on cognitive function, quality of life, and mood [42]. Assessments were performed at baseline and 12, 24 (end of treatment), and 30 (end of washout) weeks. Of the 34 subjects enrolled, 23 (68%) completed 12 weeks of treatment and 19 (56%) completed the full 24 weeks of treatment. Five subjects discontinued treatment due to toxicity (4 with GI symptoms and 1 with intracranial bleed), 5 subjects discontinued treatment because of no perceived benefit, and 5 subjects discontinued treatment due to intercurrent illness. Global cognitive function was measured by the MMSE. Executive function was measured by the TMT B. Attention and concentration were measured by the TMT A and Digit Span Test. Visual-constructional skills and figural memory were measured by the ROCFT. Verbal fluency was measured by the F-A-S Test. Verbal learning and memory were assessed by the California Verbal Learning Test Part II (CVLT-II). At baseline, cognitive impairment was clinically significant, as mean scores on attention, concentration, memory, and executive function were >1.5 SDs worse than in an age-matched normative sample. At 24 weeks, executive function on the TMT B, attention/concentration on the TMT A, and intermediate and delayed recall non-verbal memory on the ROCFT all significantly improved. All other cognitive function tasks did not change at 24 weeks. Quality of life, according to the Functional Assessment of Cancer Therapy brain and physical subscales and distressed mood on the Profile of Mood States, both significantly improved at 24 weeks compared to baseline.

In our laboratory, we investigated the effect of commercial formulations Ginkgo Synergy (120 mg/day *Ginkgo biloba* leaf, 80 mg/day *Ginkgo biloba* whole extract, and other compounds) plus 700 mg/day of choline (*n*=33) and OPC Synergy plus Catalyn (*n*=31) versus placebo (*n*=33) in a 6-month, randomized, double-blind trial on cognitive and immune functioning among English-speaking, non-smoking, healthy older adults with no cognitive deficits [43]. A neuropsychological battery, including the Stroop Test, TMT A and B, Controlled Oral Word Association (COWA), Hopkins Verbal Learning, MMSE, and Digit Symbol, was administered at baseline and 3 and 6 months follow-up to assess cognitive functioning. According to time on the TMT B, the Ginkgo Synergy plus choline arm showed improvement from baseline to 3 months follow-up. On the COWA Trial-S, the scores significantly increased for the Ginkgo Synergy plus choline arm from baseline to 6 months follow-up, and for the OPC Synergy plus Catalyn arm from baseline to 3 months follow-up. No serious adverse events were recorded. Thus, this study showed modest positive effects of the combination of *Ginkgo biloba* plus choline on isolated cognitive functioning scales.

Solomon *et al*. investigated the effect of 40 mg/day of *Ginkgo biloba* (*n*=115) compared to placebo (*n*=115) on cognitive functioning for 6 weeks among 230 community-dwelling healthy participants 60 years of age and older [44]. The results of the study did not show any improvements in learning, memory, attention, or concentration between the 2 groups at the follow-up assessment.

3.2.3.2 *Ginkgo biloba* extract (EGb 761)

In a study of adults aged 55 – 86 years without a history of cognitive dysfunction (*n*=48), subjects were randomized for 6 weeks to 180 mg/day EGb 761 (*n*=24) or placebo (*n*=24) to investigate its short-term efficacy for enhancing cognition [45]. Outcome measures included the MMSE, the Stroop Test, TMT A and B, and the WMS - Logic Memory I and II and Visual Reproduction I and II. The EGb761 group showed significantly more improvement at 6 weeks compared to placebo on the Stroop Test, which is a measure of the speed of processing abilities. No other assessments changed at the end of the intervention for either group. More participants receiving EGb 761 rated their ability to remember at the end of treatment as “improved” compared to placebo.

In a study by the same investigators in the previous study, community-dwelling volunteers aged 60 years and older (*n*=262) were randomly assigned to EGb 761 (60 mg 3 times/day; *n*=131) or placebo (*n*=131) for 6 weeks, to investigate the impact of *Ginkgo biloba* treatment on neuropsychological functioning of cognitively intact older adults [46]. Outcome measures included the Buschke Selective Reminding Test (SRT), the WAIS-III Block Design and Digit Symbol Coding tests, and the WMS-III Faces I and II. Subjects receiving EGb 761 treatment had significantly greater improvement on the SRT tasks of delayed free recall and recognition compared to placebo at the end of the intervention. On the WMS-III Faces II, the placebo group performed significantly better at baseline, but delayed recognition on this assessment was performed better by the EGb 761 group at 6 weeks. More subjects in the treatment group rated their overall ability to remember as “improved” on the subjective follow-up questionnaire. No other significant differences were detected between groups at the end of the intervention.

In a study of middle-aged healthy volunteers aged 45 – 56 years (*n*=188), subjects were randomized for 6 weeks to receive 240 mg/day of EGb 761 (*n*=94) or placebo (*n*=94), to determine the effects on memory performance in a demanding standardized free recall paradigm (list of appointments) and a less demanding standardized recognition test (driving route) [47]. At 6 weeks in the EGb 761 group, the number of correctly recalled appointments (immediate and delayed recall) significantly increased from baseline, as compared to placebo. The EGb 761 treatment group also had improved quality of immediate and delayed recall (ratio of false-to-correct) after 6 weeks, as compared to placebo. The change from baseline to 6 weeks between the treatment and placebo groups on the less demanding recognition test was not significantly different.

In a long-term, multi-site study, and community-dwelling adults aged 72 – 96 years who had normal cognitive function or MCI (*n*=3,069) were randomized to receive 120 mg 2 times/day of EGb 761 (*n*=1,545) or placebo (*n*=1,524) for a median of 6.1 years, to determine if *Ginkgo biloba* slows global or domain-specific cognitive function decline in older adults [48]. Outcomes were the rates of change for the Modified MMSE (3MSE), the ADAS-cog, and neuropsychological domains of memory, attention, visual-spatial construction, language, and executive function. Memory was assessed by the CVLT and recall from the modified ROCFT. Visual-spatial construction was assessed by the copy condition of the ROCFT and the modified WAIS-R Block Design. Language was assessed by a 30-item Boston Naming Test and semantic verbal fluency. Attention and psychomotor speed were measured by WAIS-R Digit Span and the TMT A. Executive function was measured by the TMT B and the Stroop Test. Outcomes (3MSE and ADAS-cog) were administered every 6 months through the first 4 years of follow-up, and then the ADAS-cog was administered annually after that. Rates of annual decline in the neuropsychological domains did not significantly differ between groups. Rates of change in the 3MSE and ADAS-cog varied based on initial cognitive status, but no differences were found between groups. Thus, long-term treatment with the *Ginkgo biloba* extract did not attenuate cognitive decline in older adults with normal cognitive function or MCI.

In a study of outpatients with pre-senile and senile AD type dementia and multi-infarct dementia (*n*=205), subjects were randomized for 24 weeks to receive either 240 mg/day of EGb 761 (*n*=106) or placebo (*n*=99) [49]. Cognitive function was initially measured using the Syndrom-Kurztest Cognitive Battery (SKT) and was converted to an estimated ADAS-cog. The *Ginkgo biloba* treatment group improved by 2.1 and 2.7 points on the SKT and estimated ADAS-cog, respectively, at 24 weeks, whereas the placebo group improved by 1.0 and 1.3 points on the SKT and estimated ADAS-cog, respectively. These 2 differences were significant between groups. A change in the estimated ADAS-cog score of at least 4 points is defined as a clinically significant treatment response. The response rate in the *Ginkgo biloba* group was 35%, and it was 19% in the placebo group, which was significantly different. When a 2-point improvement in the estimated ADAS-cog score was used to define treatment response (an improvement that is less clinically significant, but still important to patients), 61% of the *Ginkgo biloba* group were responders, and 37% of the placebo group were responders, which was also statistically significant. The *Ginkgo biloba* group had a significantly different improvement in the Clinical Global Impression (CGI) of change (item 2). On the other hand, *Ginkgo biloba* extract treatment had no significant effect on cognitive functioning as measured by the ADAS-cog and participant and caregiver-related quality of life, in another study where subjects with mild to moderate dementia (*n*=176) were randomized for 6 months to receive 120 mg/day of EGb 761(*n*=88) or placebo (*n*=88) [50].

In a study of patients with mild to moderate AD aged 50 – 80 years (*n*=76), participants were randomized for 24 weeks to receive either 160 mg/day of EGb 761 (*n*=25), 5 mg/day of donepezil (*n*=25), or placebo (*n*=26) as compared to donepezil and placebo, to investigate its efficacy on cognitive function according to the SKT and the MMSE [51]. Changes in overall patient condition and therapeutic efficacy were assessed using the CGI. The MMSE improved non-significantly after 12 weeks in the EGb 761 and donepezil groups. The placebo group worsened after 12 weeks, but function was not significantly different from baseline. SKT scores significantly improved in the EGb 761 and donepezil groups, as compared to placebo. The placebo group had a significantly worse SKT score after 12 weeks compared to baseline. The SKT scores were not different between the EGb 761 and donepezil groups at 24 weeks. The CGI scores significantly improved for the EGb 761 and donepezil groups at 24 weeks compared to baseline. Thus, *Ginkgo biloba* treatment had similar clinical efficacy to donepezil in the treatment of AD on cognitive functioning and global improvement.

Napryeyenko *et al*. randomized 400 subjects (≥50 years of age) with dementia (218 with probable AD or possible AD with cerebrovascular disease and 182 with probable vascular dementia) to assess the effect of 22 weeks of EGb 761 of 240 mg/day versus placebo on the Short Syndrome Test, a cross-culturally validated cognitive test battery, the Neuropsychiatric Inventory, the Verbal Fluency Test, the Clock-Drawing Test, the Hamilton Rating Scale for Depression, and the Gottfries-Bråne-Steen Scale [52]. Those with AD and vascular dementia taking the *Ginkgo biloba* extract had higher Short Syndrome Test total scores compared to the participants on placebo, whose scores decreased. Significant drug-placebo differences were noted for all other outcome variables, with no differences between AD and vascular dementia subgroups. Adverse events were higher for the placebo group.

In a study of patients with mild to moderate dementia (AD or vascular dementia) associated with neuropsychiatric symptoms (*n*=410), subjects were randomized for 24 weeks to receive 240 mg/day of EGb 761 (*n*=205) or placebo (*n*=205) to determine its effect on the SKT and the Neuropsychiatric Inventory score [53]. On the SKT total score, subjects in the treatment group improved by 2.2+3.5 points, while those receiving placebo improved only by 0.3+3.7 points, and this difference was statistically significant. On the Neuropsychiatric Inventory composite score, subjects in the treatment group improved by 4.6+7.1 points compared to the placebo group that improved by 2.1+6.5 points, and this difference was also statistically significant. Several secondary outcomes, including the ADCS CGI of change, the Verbal Fluency Test, the ADL International Scale overall mean score, the Dementia Quality of Life Instrument-Proxy total score, and the 11-point box scale for dizziness, were significantly better in the treatment group compared to placebo at the end of the intervention.

In a study of patients with MS, subjects (*n*=121) were randomized for 12 weeks to receive 240 mg/day of EGb 761 (*n*=61) or placebo (*n*=60), to investigate its effects on cognitive functioning [54]. The Stroop Test, CVLT-II, COWA, and the Paced Auditory Serial Addition Task were used as screening and outcome assessments. Treatment with EGb 761 did not improve cognitive performance at 12 weeks compared to baseline or compared to placebo.

In a study of patients with MCI, subjects (*n*=160) were randomized for 24 weeks to receive EGb761 240 mg/day (*n*=80) or placebo (*n*=80) to determine its effects on cognition and neuropsychiatric symptoms [55]. Cognition and global ratings of change were measured by the TMT A and B. The treatment group had higher scores on the TMT A and B and had better outcomes according to the caregiver’s global impression of change at 24 weeks. The treatment group also had a significant improvement on the State-Trait Anxiety Inventory and a trend toward a better Geriatric Depression Scale score at 24 weeks.

In a study of healthy male and female elderly volunteers with subjective memory impairment (*n*=61), subjects were randomized for 60 days to receive either 240 mg/day of *Ginkgo biloba* extract EGb 761 (*n*=31) or placebo (*n*=30) [56]. The primary outcomes were task-set switching, response inhibition, delayed response, prospective memory, task-related fMRI blood-oxygen-level-dependent signals, and the Trier Social Stress Test. The *Ginkgo biloba* treatment group showed significantly improved cognitive flexibility compared to placebo according to decreased task-set switch costs, which was not due to differences in depression symptoms, an unequal ratio of genders between the groups, or because of changes in brain activation, indicating a low cost of neural systems/resources. Thus, this result was likely due to increased cognitive processing efficiency. A trend was also noted for improved response inhibition in the *Ginkgo biloba* treatment group, as demonstrated by Go-NoGo-task reaction times corrected for error rates. These 2 findings (better cognitive flexibility without changes in brain activation and improved response inhibition) are compatible with mild enhancement of prefrontal dopamine, but the effects of *Ginkgo biloba* on prefrontal dopaminergic functions require further research. No other outcomes significantly changed at the end of the intervention.

In a study of outpatient vascular MCI patients (*n*=62), *Ginkgo biloba* was used as a control compared to Pushen (a capsule that contains many Traditional Chinese Medicine ingredients) on measures of cognitive function [57]. Subjects were randomized but not blinded for 12 weeks to treatment with Pushen (1.8 mg, 3 times/day; *n*=30) or *Ginkgo biloba* (19.2 mg/day of EGb 761; *n*=32). Cognitive outcomes were the MMSE, the MoCA, and the Subjective Memory Loss Rating Scale scores. At 12 weeks, the MMSE score of the Pushen group was significantly higher than baseline, but not significantly different from the *Ginkgo biloba* group. The MMSE score modestly improved at 12 weeks in the *Ginkgo biloba* group. The MoCA score and the delayed recall item score were significantly higher at week 12 compared to baseline for both groups. The Subjective Memory Loss score and the cognitive function “forgetting acquaintance’s name” were higher at 12 weeks compared to baseline in the Pushen group. The *Ginkgo biloba* group improved in the overall Subjective Memory Loss score but performed significantly worse than the Pushen group for “forgetting acquaintance’s name” at 12 weeks. Thus, the results suggest that cognitive function improved comparably between Pushen and *Ginkgo biloba*.

Overall, *Ginkgo biloba* and its extract EGb 761 show promise as a clinically significant compound for improving cognitive function in cognitively normal adults and in those with MCI or varying degrees of dementia. In general, these improvements are without adverse effects. Nonetheless, some inconsistencies in the findings of the studies can be due to dose, plant form, extract strength, and other associated production and quality factors in addition to differences in study design and population.

#### 3.2.4. *Ginseng - Panax ginseng* C.A. *Mey and Panax* (American) *quinquefolius*

Ginseng is one of the most widely consumed herbal products in the world and has long been used for its apparent medicinal and performance-enhancing properties. The root extract is highly regarded in Asian herbal medicine due to its reputation in promoting longevity, acting as an adaptogen, and serving as an adjunct treatment in numerous conditions including diabetes, cardiovascular disease, and inflammatory disorders [196]. The proposed beneficial effect in cognitive disorders may be mediated through disease-modifying pathways such as reduction in amyloidogenesis, inflammation, or neurotoxicity. Mechanistically, the ginsenosides Rg_3_, Rh_1_, Rh_2_, Rb_1_, Rd, Rg_2_, and Rb_3_ and the aglycones PPD and PPT have shown encouraging results in animal and cell-based studies [197]. Likewise, the metabolite Compound K has shown neuroprotective effects in non-human trials. However, pharmacological and human clinical trial data in the use of ginseng have been largely equivocal and limited by poor methodological quality of studies [197].

In a double-blind, placebo-controlled, parallel-group, multi-center trial of 279 healthy middle-aged volunteers, 2 dosing regimens (160 mg 2 times/day or 320 mg/day) of a capsule of a standardized extract of 60 mg *Ginkgo biloba* (GK501), and a standardized extract of 100 mg *Panax ginseng* were administered for 14 weeks to assess various aspects of cognitive function [58]. At baseline and weeks 4, 8, 12, and 14, the volunteers performed attention and memory tests from the Cognitive Drug Research computerized cognitive assessment system prior to morning dosing and again at 1, 3, and 6 h later. A quality index for working and long-term memory significantly improved by 7.5% in response to the *Ginkgo biloba*/*Panax ginseng* combination. The memory improvement occurred throughout the 12-week dosing period and remained after a 2-week washout.

In a double-blind, placebo-controlled clinical trial, and 90 Korean volunteers with MCI were randomized for 6 months to either 3 g/day of *Panax ginseng* powder (*n*=45) or starch placebo (*n*=45) to determine its cognition-enhancing effects [59]. Cognition was assessed using the Korean Mini-Mental Status Examination (K-MMSE), the ROCFT for immediate and 20-min delayed recall, the Korean IADL, and the Seoul Neuropsychological Screening Battery. The *Panax ginseng* group improved significantly on both immediate and 20-min delayed recall tests on the ROCFT compared to placebo. No serious adverse events were noted. These results suggest that *Panax ginseng* may be beneficial in improving visual memory function but does not appear to influence verbal memory.

Another 12-week open-label randomized trial sought to examine the efficacy of Korean red ginseng as an adjuvant therapy to conventional anti-dementia medications in patients with AD [60]. Sixty-one patients were randomized to low-dose Korean red ginseng (4.5 g/day; *n*=15), high-dose Korean red ginseng (9 g/day; *n*=15), or control (*n*=31), and cognitive function was assessed using the ADAS, the K-MMSE, and the Clinical Dementia Rating (CDR) scale. Patients in the high-dose ginseng group showed significant improvements on the ADAS and CDR after 12 weeks of treatment compared to controls. However, the ADAS-non-cog and MMSE scores were not significantly different between the 2 ginseng groups and the controls.

The same group investigated different doses of ginseng in another open-label study of AD patients [61]. Forty patients were randomized into one of 3 different dose groups (1.5 g/day, 3 g/day, or 4.5 g/day; *n*=10 each) or the control group (*n*=10), and the ADAS and MMSE were used to assess cognitive function for 24 weeks. Patients in the highest dose group (4.5 g/day) showed statistically significant improvements in the ADAS-cog, ADAS-non-cog, and MMSE scores at 12 weeks and at the 24-week follow-up, while the lower dosing and control groups did not exhibit significant improvement over the course of the study.

A hydroponically cultivated red *Panax ginseng* Meyer root preparation (HRG80) and a traditionally harvested white *Panax ginseng* standard preparation (PGS) were evaluated for their effects in a number of tests including the attention d2 test for cognitive function, a computerized memory test, and the perceived stress score [62]. The effects of HRG80 and PGS were studied in this 3-arm, randomized, double-blinded, and placebo-controlled crossover trial of 50 healthy subjects for 2 weeks. Subjects either took 2 capsules/day (418 mg each) of HRG80 (*n*=17), 2 capsules/day (384 mg each) of PGS (*n*=16), or placebo (*n*=17). A statistically significant interaction effect between time and treatment was observed in the attention d2 and memory tests, indicating that HRG80 treatment was more beneficial than the placebo. The effect of PGS was not statistically significant in the attention test, although it was better than placebo. A significant difference was seen between the effects of HRG80 and PGS in attention, both after a single dose (day 1) and after repeated administrations on days 5 and 12 of treatment. Overall, HRG80 treatment with a higher content of ginsenosides was superior to PGS and placebo, according to attention and perceived-stress scores after single and repeated administrations for 5 and 12 days, while memory scores during both HRG80 and PGS supplementation were superior to placebo at the same time points.

In an open-label study of patients with AD, 97 participants were randomized to a 4.5 g/day *Panax ginseng* powder (*n*=58) or a control group (*n*=39) for 12 weeks [63]. Cognitive performance was measured using the MMSE and ADAS during 12 weeks of ginseng treatment and at 12 weeks after ginseng discontinuation. Ginseng treatment showed improvements in the ADAS-cog and MMSE scores that continued up to 12 weeks, with declines to control levels after discontinuation of ginseng. These results suggest that ginseng may improve cognitive performance in AD patients, although ginseng was not studied against placebo.

In a double-blind, crossover study, healthy young adults (*n*=32) were assessed on acute neurocognitive effects after receiving, in random order, either 100, 200, or 400 mg of American ginseng (Cereboost composed of *Panax quinquefolius* standardized to 10.65% ginsenosides) or a placebo [64]. Treatment occurred over 4 separate days, with a 7-day washout period in between each administration. Cognitive function was measured at 1, 3, and 6 h following ginseng administration with the Computerized Mental Performance Assessment System battery, which was developed to include tests sensitive to nutritional manipulations. A significant improvement in working memory was found. Corsi block performance, a measure of spatial span and speed of response, also improved for all doses at all testing times. Differential effects of all doses on other working memory tasks were noted across the testing day, while choice reaction time accuracy and calmness were significantly improved after the 100 mg dose. Thus, ginseng appears capable of improving working memory in healthy adults.

HT1001, a proprietary North American ginseng extract, was investigated for its effects on working memory over 4 weeks in a double-blind, placebo-controlled study of 64 individuals with stable schizophrenia [65]. Verbal working memory and visual working memory were assessed at baseline and at the end of the treatment period using the Letter-Number Span Test and Visual Pattern Test. Symptoms and medication side effects were also assessed. HT1001 treatment (*n*=32) led to improvement in visual working memory compared to placebo (*n*=32). Interestingly, the HT1001 treatment group after 4 weeks also showed a decrease in extrapyramidal symptoms, while no change in extrapyramidal symptoms was noted in the placebo group. The improvement in working memory and reduction in extrapyramidal effects warrant further investigation of HT1001 as an adjunct therapy in schizophrenia. A reduction in medication-related side effects could greatly improve the quality of life and functional status of individuals with schizophrenia.

Overall, although many individual studies suggest that ginseng has potentially beneficial effects on cognition, further larger scale, randomized, and placebo-controlled trials are needed to determine its role in enhancing cognitive function. Importantly, ginseng may improve cognitive function in AD patients, but more subjects over a longer period of study are needed to corroborate the current findings. In addition, the positive results are difficult to generalize because of differences in dose and type of ginseng, which further study could elucidate.

#### 3.2.5. Lion’s mane mushroom (*Hericium erinaceus*)

Many mushrooms have been historically used to treat ailments [198], and lion’s mane mushroom is used for both culinary and medicinal purposes, showing the ability to enhance the brain through neurological growth [199]. In animal models, its active compounds have demonstrated the ability to delay neuronal death in neurodegenerative diseases such as ischemic stroke, Parkinson’s disease, AD, and depression, and it has been shown to promote nerve regeneration and functional recovery in neuropathic pain or presbycusis [199]. In addition, lion’s mane has been shown to prevent the loss of spatial short-term and visual recognition memory induced by Aβ25-35 in mice [200]. Similarly, it has been shown *in vitro* that lion’s mane fruiting body extract induces neurite outgrowth of neuron cells NG108-15 and PCI2 cells, promotes nerve growth factor (NGF) mRNA expression, and modulates the secretion of NGF from 1321N1 human astrocyte cells [201]. Although the preclinical data are promising, the results from clinical trials are currently limited.

Lion’s mane was assessed for its efficacy on cognitive function with the Revised Hasegawa Dementia Scale (HDS-R) in a double-blind, placebo-controlled trial of Japanese men and women 50 – 80 years old with MCI (*n*=30) [66]. Subjects were randomized for 16 weeks to either four 250 mg tablets containing 96% of lion’s mane dry powder 3 times/day (*n*=15) or placebo (*n*=15). At weeks 8, 12, and 16, the lion’s mane group showed significantly higher scores on the HDS-R compared to placebo. The improvement was greater with increasing duration of the lion’s mane intake. However, after 4 weeks of termination of treatment, the HDS-R significantly declined. No side effects of the treatment were observed throughout the study. Hence, the results indicated that treatment with lion’s mane could lead to improved cognitive function in older people with MCI with no side effects.

In a more recent double-blind, placebo-controlled study assessing the effect of lion’s mane on cognitive function, healthy older adults over 50 years of age (*n*=31) were randomized for 12 weeks to either 4 times/day dose of 0.8 g of lion’s mane (3.2 g/day total; *n*=16) or placebo (*n*=15) [67]. Cognitive function was assessed at baseline and 6 and 12 weeks using the MMSE, the Benton visual retention test, and the Standard verbal paired-associate learning test. The results showed significant improvement in cognitive function in the lion’s mane group compared to placebo on the MMSE at 12 weeks, but no other assessments were significantly different. Thus, in these 2 clinical trials, lion’s mane showed improvements in cognitive function within 4 months in both healthy older adults and in older adults with MCI. These studies also noted no adverse effects, suggesting that lion’s mane is a safe treatment.

#### 3.2.6 *Rhodiola rosea L*.

*Rhodiola rosea* is a plant found in the mountainous regions of the Arctic, Europe, Asia, and North America with roots that have long been used in traditional medicine [202]. The plant has been used in the treatment of anxiety, depression, and fatigue and offers promise in improving cognition and mental performance due to its neuroprotective properties [203]. The plant’s roots contain biologically-active compounds such as flavonoids and glycosides (e.g., salidroside) that help the body resist biological stressors and avoid damage, hence earning its title as an adaptogen [204]. Several mechanisms may explain *Rhodiola rosea*’s possible neuroprotective and cognition-promoting properties. The plant may interfere with the secretion of stress-response compounds such as the hormone cortisol within the hypothalamic-pituitary-adrenal system [205]. In addition, *Rhodiola rosea* may interact with p-JNK, which are protein kinases involved in stress signaling pathways implicated in Aβ accumulation associated with AD [206]. Finally, *Rhodiola rosea* has antioxidant properties and may act as a free-radical scavenger to combat oxidative stress that may be associated with AD.

In a placebo-controlled double-blind study, the effect of a daily low-dose regimen of 170 mg SHR-5 *Rhodiola rosea* extract (4.5 mg of salidroside) was tested versus placebo on the mental performance of healthy young physicians aged 24 to 35 (*n*=56) with nonspecific fatigue during night shifts [68]. Subjects were randomly assigned to group A (*n*=26) that received a daily dose of SHR-5 for 2 weeks or group B (*n*=30) that received a daily dose of placebo for 2 weeks. After a 2-week wash-out period, subjects were crossed over to receive the treatment that they had not received before for 2 weeks (i.e., group A received the placebo and group B received the SHR-5). Five tests were used to measure visual and audial perception speed, attention capacity, and short-term memory as indicators of fatigue. The result was calculated as a fatigue index, which was the ratio of the test score before night duty to the test score after night duty multiplied by 100. The results revealed that the total fatigue index was significantly improved after 2 weeks of taking the SHR-5 dose when compared to placebo, with no reported adverse effects.

The effect of *Rhodiola rosea* on the stress, anxiety, mood, sleep, sleepiness, and cognitive function of healthy, mildly anxious university students (ages 18 to 35; *n*=81) was assessed in a randomized open-label trial [69]. A group (*n*=40) received 200 mg/day of Vitano (Rosalin (WS 1375) dry extract from *Rhodiola rosea* root) 2 times/day (30 min before breakfast and 30 min before lunch) for 14 days. The control group (*n*=41) received no treatment. Stress, anxiety, mood, sleep, and sleepiness levels were all self-reported through a questionnaire. Simple reaction time, choice reaction time, sustained assessment to response test, and symbol digit processing were assessed through cognitive tests. The study had 4 phases: (1) a baseline questionnaire and cognitive tests, (2) dosing followed by the questionnaire and cognitive tests 4 h later, (3) a questionnaire and cognitive tests at 7 days, and (4) a questionnaire and cognitive tests at 14 days. Self-reported anxiety and stress were significantly reduced in the treatment group by 14 days. The treatment group reported significantly lower levels of anger, depression, and confusion and significantly improved overall mood. Sleep or sleepiness was not significantly different between the control and treatment groups. Cognitive performance did not change for either group. The results demonstrate that Vitano was effective at reducing anxiety and stress and improving mood in healthy university students but did not significantly improve cognitive performance compared to the control. Further research in this area would benefit from utilizing a placebo control group.

In another study, 12 weeks of *Rhodiola rosea* extract combined with a vitamin and mineral supplement (Vigodana) was assessed on the cognitive function of adults aged 50 to 89 with physical and cognitive disabilities (*n*=120) [70]. Vigodana contains vitamins E, B6, and B12, folate, magnesium, and *Rhodiola rosea* root extract. All subjects had a self-perceived cognitive or physical disability and had total error scores ≥8 on the Orientation-Memory-Concentration Test. Subjects with AD, Creutzfeldt-Jakob disease, Parkinson’s, brain trauma, and cerebral tumor were excluded from the study. Group 1 (*n*=60) took 2 capsules after breakfast, and group 2 (*n*=60) took 1 capsule after breakfast and 1 after lunch, and subjects were assessed at baseline, 6 weeks, and 12 weeks. Physical and cognitive performance was assessed through a digit connection test, a 4-point rating scale, and evaluation of efficacy and tolerability by the patient and a physician. The digit connection test evaluated mental vitality by assessing the time it took subjects to complete the test. The 4-point rating scale was used to assess symptoms of physical impairment such as exhaustion, decreased motivation, daytime sleepiness, decreased libido, and sleep disturbances and symptoms of cognitive impairment such as concentration impairment, forgetfulness, memory deficiency, susceptibility to stress, and irritability. Results revealed that both groups had significantly better physical performance at 12 weeks compared to baseline and highly significant improvement in cognitive performance compared to baseline. Group 1 showed consistently higher improvements in both physical and cognitive performance by week 12 when compared to group 2, indicating that the dosage for group 1 seemed to be more effective than that of group 2. Physicians assessed the efficacy of the supplement as “very good” or “good” for 81% of patients, and 80% of patients assessed the efficacy of the supplement as “very good” or “good” for themselves. Thus, this study demonstrated that Vigodana seemed to improve the cognitive and physical performance of subjects at 12 weeks compared to baseline with no adverse effects. A limitation of this study was that it was not placebo-controlled, which would be a next step in this line of research.

The effect of a dose of ADAPT-232 (containing a standardized extract ratio 2.8:1 *Rhodiola rosea*, 1.4:1 *Schisandra chinensis* (Turcz.) Baill., and 10.5:1 *Eleutherococcus senticosus* Maxim) on the mental performance of healthy females aged 20 to 68 (*n*=40) with chronic stress was assessed in a randomized, double-blind, placebo-controlled, and parallel-group trial [71]. The subjects were randomly divided into a group (*n*=20) that received a single dose of 270 mg of ADAPT-232 or another group (*n*=20) that received a placebo dose. The d2 Test of Attention and the Stroop Test were used to assess mental performance during stressful cognitive tests (specifically their attention, speed, and accuracy). The first day was the baseline assessment. On the second day, subjects were tested again once in the morning and once in the afternoon. On the third day, subjects took their doses of either ADAPT-232 or placebo and then were assessed 2 h later. The treatment group performed significantly better than the control group on attention, speed, and accuracy on the d2 test. No major adverse effects were reported, although a few instances of sleepiness and cold extremities were observed in both the treatment and placebo groups. This study suggests that ADAPT-232 was effective at improving subjects’ mental performance and attention, speed, and accuracy under stressful conditions. It is unclear how much *Rhodiola rosea* is responsible for this improvement in performance, as ADAPT-232 contains a combination of plant compounds.

*Rhodiola rosea* offers promise as a treatment for anxiety, stress, and fatigue in healthy adults and as a possible enhancer of mood, mental performance, and cognition. *Rhodiola rosea* also appears to be safe for human consumption with little to no adverse effects. However, further research should be conducted regarding its effect on the cognition of older adults with cognitive decline and neurodegenerative diseases such as AD, and trials should be placebo-controlled and include larger sample sizes.

#### 3.2.7. Rosemary (*Rosmarinus officinalis L*.)

Rosemary has long been theorized to stimulate the brain and assist in memory, with this thought dating back to ancient Greece [207]. Studies have shown that rosemary extract can scavenge reactive oxygen species [208] and exert a neuroprotective effect on dopaminergic neurons [209]. Therefore, it has been investigated as a treatment to improve cognitive function.

In a crossover study, 28 healthy elderly individuals (65 – 90 years of age) received in random order one of 4 doses of dried rosemary (750, 1,500, 3,000, or 6,000 mg) or placebo on 5 separate 1-day treatment sessions every week for 5 weeks [72]. Cognitive performance was assessed immediately after consumption and at 1, 2.5, 4, and 6 h post-consumption. The 750 mg dose resulted in a significant improvement in speed of memory compared to placebo, while 6,000 mg resulted in impairment. Compared to baseline, all doses resulted in significant impairment, other than the 750 mg dose. Continuity of attention was significantly impaired compared to placebo at every dose other than 750 mg, and quality of working memory was significantly impaired compared to placebo at every dose other than 3,000 mg. Subjective feelings of alertness were significantly improved for the 750 mg dose, and alertness was significantly decreased for the 6,000 mg dose, compared to placebo. Therefore, the lower dose of rosemary appeared to improve speed of memory and alertness, preventing time-related performance decrements, which may be attributed to fatigue. In contrast, the higher doses negatively affected performance and subjective feelings of alertness.

In another study, 76 young adults with low energy were randomized to a 1-time consumption of either a dose of 1.7 g of rosemary (*n*=26), black pepper (*n*=26), or placebo (*n*=24), measuring task performance before and 60 and 90 min after consumption [73]. The results showed that rosemary decreased mental fatigue on a visual analog scale at 60 min and reduced false alarms during the primary cognitive task at 90 min, although these changes were statistically insignificant and transient. Thus, rosemary does not induce consistent acute improvements in cognitive performance, at least at the dose used in this study.

In a study of healthy adults, 80 subjects were randomized to receive either 250 mL of water infused with rosemary (*n*=40) or plain water (*n*=40) with a 20-min absorption period before cognitive assessment [74]. Those who drank rosemary had small to moderate beneficial effects for performance on several tasks: The Corsi blocks mean span length, serial threes and serial sevens correct responses, RVIP correct responses and errors, and immediate and delayed word recall. Curiously, the effect of rosemary on fatigue of a visual analog scale was slightly negative. Those who received rosemary also had a significant increase in deoxygenated hemoglobin compared to placebo. However, this effect became insignificant with a Bonferroni correction. The overall main effect of time on deoxygenated hemoglobin was also significant, as it decreased in the middle of the testing period and increased toward the end. Therefore, treatment with rosemary water improved cognitive task performance and cerebral oxygen extraction. However, the performance on several of the cognitive tasks was unchanged, putting into question whether the noted improvements were a direct result of the increases in oxygen extraction, warranting further exploration of this relationship.

In addition to oral consumption, the effects of aromatherapy rosemary have been investigated as well. Aroma inhalation with rosemary has been found to stimulate arousal, producing effects on both subjective mood and EEG [210]. It is well established that arousal affects task performance, following the inverted-U curve of performance versus arousal [211].

Therefore, 144 healthy individuals were randomized to receive a 1-time administration of either ambient aromatherapy with 4 drops of rosemary essential oil (*n*=48), lavender essential oil (*n*=48), or an odorless control (*n*=48), to assess subjective mood and cognitive task performance before and after treatment [75]. The rosemary treatment had significantly higher scores on a secondary memory subfactor (indicating better accuracy during memory-related tasks) and subjective feelings of alertness and contentedness compared to control. However, the control treatment had significantly quicker responses (speed of memory factor and speed of attention factor) than the rosemary treatment. These results support the concept of ambient aroma altering mood and significantly affecting aspects of cognitive performance. However, rosemary may cause a speed-accuracy trade-off, where greater accuracy is accompanied by slower responses in comparison to control. Furthermore, rosemary appeared to improve performance on more demanding tasks related to memory consolidation and retrieval, but not less demanding attentional tasks. It is possible that rosemary aroma increases arousal excessively for less demanding tasks but optimally for those that are more demanding. Thus, it appears rosemary aromatherapy may improve cognitive performance, but its effectiveness greatly depends on the nature of the task.

The dose of rosemary greatly impacts its effects on cognitive function, and it has even been shown to impair performance between 1,500 and 6,000 mg/day. Rosemary was also shown to have a deleterious effect on speed of memory. Nonetheless, other research shows that rosemary can be beneficial for cognitive function, creating overall conflicting results that are difficult to interpret, questioning its clinical significance. Thus, supplementation beyond normal culinary use may not be advantageous for cognitive function.

#### 3.2.8. Saffron (*Crocus sativus L*.)

Saffron is a spice derived from the dried stigmata of the plant *Crocus sativus* (from the Iridaceae family). The stigmata of *Crocus sativus* have been used for centuries in traditional medicine, and saffron has been shown to have 3 main metabolites: picrocrocins, safranal, and crocins [212].

Based on what is currently known about saffron, it may be beneficial for AD patients mainly due to its antioxidant properties. However, its crocins, water-soluble carotenoids that are the primary metabolites involved in saffron’s memory and cognition-enhancing effects, were shown to be effective in stopping Aβ plaque formation, crucial for preventing AD and perhaps for treatment of those with the disease [213,214]. Crocins were also shown in separate studies to prevent the formation of neurofibrillary tangles [214-216]. The findings from these 3 studies are important because Aβ aggregation and neurofibrillary tangles are 2 of the main histopathological findings in AD. Another proposed mechanism of action for saffron’s ability to prevent cognitive decline is through a moderate (up to 30%) inhibitory activity on AChE [217,218]. Finally, saffron has also been shown to be as effective as fluoxetine and imipramine in the treatment of mild to moderate depression [219-221], which could be important for the treatment of mood disorders that typically go hand-in-hand with cognitive difficulties. Nonetheless, despite the potent properties of the plant and the initial promising findings, saffron has only been evaluated so far in a handful of clinical trials [222].

In a study conducted in mild to moderate AD patients, subjects (*n*=46) were randomized for 16 weeks to receive either 30 mg/day of saffron (*n*=23) or placebo (*n*=23) to determine its effects on cognitive function with the MMSE, ADAS-cog, and the CDR scale-sums of boxes (CDR-SB) at baseline and every 2 weeks [76]. At the end of 16 weeks, the saffron group showed statistically significant improvements in cognitive function according to the ADAS-cog and CDR-SB compared to the placebo group, and no severe adverse effects were reported. In a follow-up trial conducted by the same group of researchers, 54 patients with mild to moderate AD were randomized for 22 weeks to receive either 30 mg/day of saffron (*n*=27) or 10 mg/day of donepezil (*n*=27), an FDA-approved AChEI [77]. The same assessment battery was administered every 2 weeks as in the previous study. The ADAS-cog and CDR-SB improved similarly for both groups, and no serious adverse effects were noted throughout the study. Thus, saffron may offer similar short-term improvement as donepezil without the possibility of untoward effects.

In a study design similar to the previous one, patients with moderate to severe AD (*n*=68) were randomized for 12 months to receive either 30 mg/day of saffron (*n*=34) or 20 mg/day of memantine (*n*=34), a commonly used drug to treat symptoms of AD [78]. Every month, subjects were evaluated with the Severe Cognitive Impairment Rating Scale and the Functional Assessment Staging in addition to adverse events. The changes from baseline to 12 months in both cognition assessments were similar for both groups, demonstrating that saffron was as effective as memantine in the prevention of further cognitive decline in these patients. In addition, the number of adverse events was not different between the 2 groups.

Finally, in another study of patients with amnestic MCI, 35 subjects were assessed and diagnosed and then were randomized for 12 months into a wait-list control no-treatment condition (*n*=18) or to saffron extract (*n*=17) [79]. Subjects were assessed at baseline and 12 months on the MoCA, the MMSE, the Geriatric Depression Scale, the Functional Rating Scale of Symptoms of Dementia for ADL, and the Neuropsychiatric Inventory. The results indicated that the subjects who received saffron had significant cognitive improvement according to the MMSE, while the wait-list control condition subjects had worsening cognitive decline. All other assessments were non-significantly different. Thus, overall saffron shows promising findings for improvement in cognitive functioning in adults with various stages of cognitive dysfunction, from MCI to severe AD. It also appears to have minimal, if any, adverse effects up to at least 1 year of consumption.

#### 3.2.9. Tart cherries (*Prunus cerasus L*.)

Tart cherries are rich in phytochemicals, such as anthocyanins [223,224]. They have been found to decrease inflammation [223] and oxidative stress [225] and improve vascular function [226]. Anthocyanins have also been found to have neuroprotective effects, as they can shield neurons from inflammation, enhance neuronal function, increase cerebral blood flow, and stimulate neurogenesis in areas of the brain necessary for cognition [227]. Impaired cerebral blood flow is hypothesized to contribute significantly to the decline in cognitive function that occurs with advancing age and neurodegenerative disease [228]. Therefore, tart cherries have the potential to be an intervention for neurodegenerative disorders, such as dementia.

In a study, 49 older adults (>70 years of age) with mild-to-moderate dementia were randomized for 12 weeks to receive either 200 mL/day of cherry juice (*n*=24) or a control juice lacking anthocyanins (*n*=25) [80]. The cherry juice group saw significant improvements in cognitive performance at 6 and 12 weeks using the category verbal fluency task, AVLT total, AVLT delayed recall, and AVLT 20-min delayed recall tasks, while the control group did not. Effect sizes were largest for the first 3 aforementioned tasks and were moderate for the last. Systolic blood pressure significantly improved and diastolic blood pressure modestly decreased in the cherry group, with no changes in the control. Therefore, consumption of anthocyanin-rich cherry juice was beneficial for multiple measures of cognitive function in individuals with AD with additional improvements in vascular function.

However, tart cherries do not appear to have immediate effects on cognitive performance compared to their long-term effects, as shown in the previous study. Young healthy adults (18 – 35 years of age; *n*=6), older adults (at least 55 years of age; *n*=5), and older adults with dementia (*n*=5) were randomized in a crossover design to a 1-time consumption of anthocyanin-rich cherry juice in one of 2 dose schemes: (1) a single 300 mL dose at 0 h or (2) 100 mL doses at 0, 1, and 2 h with cognitive tasks at baseline and 6 h [81]. Regardless of dose-timing, juice consumption did not improve acute cognition, as measured by the AVLT, pattern and letter comparison, or task-switching tests. In another crossover study, 27 middle-aged (45 – 60 years of age) individuals were randomized to a 1-time consumption of either 60 mL of tart cherry concentrate or placebo, a 1-h absorption period, and then assessment of cognitive performance, blood pressure, and prefrontal cortex cerebrovascular response at baseline and 1, 2, 3, and 5 h after consumption [82]. Those who received the tart cherry concentrate had a significant decrease in systolic blood pressure at 1, 2, and 3 h compared to placebo. This group also saw significantly higher concentrations of oxygenated hemoglobin during the 30-40 min epoch of the absorption period, compared to placebo, as well as during each period of task performance 1 h after consumption. The cherry group also had higher total hemoglobin concentrations during each period of task performance after 1 h. However, cognitive performance was not significantly different between the groups. Therefore, while tart cherry consumption resulted in acute improvements in blood pressure and in cerebral blood flow to the prefrontal cortex during task performance, this did not significantly affect performance on cognitive tasks. Regardless, the changes in cerebral blood flow have strong implications for tart cherry as an intervention for neurogenerative diseases, which warrants further investigation, perhaps using juice consumption over a long period of time and/or a greater absorption period.

#### 3.2.10. Turmeric (*Curcuma longa L*.)

The lynchpin physiologically-active compound of turmeric is curcumin [229], which has been shown to have a host of biochemical actions, including anti-carcinogenic, anti-proliferative, anti-inflammatory, antioxidant, antiviral, and antibacterial, among others, and it has been shown to act on transcription factors, enzymes, growth factors, cytokines, and neurotransmitters [230,231]. Thus, its metabolic capacity is quite diverse and extensive, but most oral applications are typically hindered by low bioavailability due to poor solubility, rapid metabolism, and swift elimination [230], making it challenging to create a therapeutic delivery method, although technical formulations are utilized, e.g., nanoencapsulation with liposomes and micelles and emulsions [232]. Because of the oral delivery limitations, the results of clinical trials have either been inconsistent, unsupportive of preclinical or animal data, or lacking in ultimate efficacy [233]. Nonetheless, extensive research demonstrates curcumin’s potential for being a key neuroprotective agent and beneficial for cognitive function in healthy adults and those with AD or dementia, based on its ability to inhibit AChE, protect against Aβ toxicity and/or limit its production, reduce the effects of oxidative stress, and decrease inflammation, among others [233-236].

In a study of 60 healthy adults aged 60 – 85 years, participants were randomized to receive either 400 mg (~80 mg curcumin) of Longvida (a solid lipid curcumin formulation; *n*=30) or placebo (*n*=30) to examine acute (1-h and 3-h post-treatment), chronic (4 weeks), and acute-on-chronic (1-h and 3-h post-treatment following chronic treatment) effects on cognitive function and mood [83]. Outcome measures were assessed at baseline and 28-day follow-up, and cognition was assessed by the Computerized Mental Performance Assessment System. At 1-h post-treatment, the curcumin group had improved performance on sustained attention (the digit vigilance task) and working memory (the serial threes subtraction task) compared to placebo. Chronic curcumin treatment significantly improved working memory (the serial threes subtraction task), mood, and fatigue compared to baseline and placebo. The acute-on-chronic treatment effect was significant for alertness and contentedness in the curcumin group. Thus, low-dose (80 mg) curcumin had significant positive acute and chronic effects on cognition and chronic effects on mood and fatigue.

In a second study by the same group of investigators, 89 healthy older adults aged 50 – 80 years were randomized for 12 weeks to receive either 400 mg (~80 mg curcumin) of Longvida (*n*=46) or placebo (*n*=43) on cognitive function and overall health [84]. Cognitive performance was assessed at baseline and 4 and 12 weeks and was measured by serial threes and serial sevens, virtual Morris Water Maze, Divided Attention Tracking Task, and Arrow Flankers Task. Compared to placebo, the curcumin group had better working memory performance on the virtual Morris Water Maze and on serial threes and serial sevens at 12 weeks. Thus, treatment with Longvida appeared to improve working memory in healthy older adults, which was consistent with the previous study.

In another study, 160 community-dwelling, cognitively healthy older adults were randomly assigned for 12 months – 500 mg 3 times/day Biocurcumax, a curcumin formulation (*n*=80), or placebo (*n*=80) for the prevention of cognitive decline [85]. Clinical and cognitive assessments were conducted at baseline and 6 and 12 months. Frequency of errors in prospective and retrospective short- and long-term memory was measured by self-report on the Prospective and Retrospective Memory Questionnaire. General cognition was assessed with the MoCA. Verbal learning and memory were assessed with the AVLT. Verbal fluency was assessed with the COWA. Perceptual motor speed was assessed with the Wechsler Digit Symbol Scale from the WAIS-R. The computerized CogState was administered and included a battery of cognitive tasks. A significant interaction effect for performance on the MoCA was related to a decline in cognitive function at 6 months in the placebo group that did not occur in the Biocurcumax group, and no other difference between groups was significant. Thus, the results of the study showed limited efficacy of Biocurcumax for delaying cognitive decline in older healthy adults.

Another study was conducted in cognitively healthy middle age and older adults (51 – 84 years of age) to determine the effects of a nanoencapsulated formulation of curcumin (Theracurmin) on cognitive function and brain Aβ and tau accumulation according to positron emission tomography scans [86]. Participants (*n*=40) were randomized for 18 months to receive 90 mg of highly bioavailable curcumin twice/day (*n*=21) or placebo (*n*=19). Cognitive assessments included verbal (SRT) and visual (Brief Visual Memory Test-Revised) memory and attention (TMT A), and positron emission tomography scans were performed in the amygdala, hypothalamus, medial and lateral temporal, posterior cingulate, parietal, frontal, and motor (reference) regions. The SRT Consistent Long-Term Retrieval, SRT Total, visual memory, and attention all significantly improved in the curcumin group compared to placebo. Brain Aβ and tau accumulation decreased significantly in the amygdala in the curcumin group compared to placebo, and while they did not change in the hypothalamus of the curcumin group, they increased in the placebo group. Thus, this is the first study documenting improvements in memory and attention in older adults after bioavailable curcumin treatment that was related to improvements in plaque and tangle accumulation in certain brain regions. Overall, the results of curcumin in clinical trials are not as conclusively impressive as in preclinical and animal studies, which point to research design issues and limitations based on curcumin’s typical low bioavailability and resultant limited therapeutic doses.

#### 3.2.11. Wild yam (*Dioscorea villosa L*.)

Diosgenin is a compound found in many species of yam, which has been found to repair axonal atrophy and synaptic degeneration, improving memory dysfunction in a mouse model of AD [237] and in normal mice [238]. Therefore, diosgenin may potentially strengthen cognitive function in both healthy adults and AD patients. In a study, 31 healthy adults (20 – 81 years of age) were randomized for 12 weeks to receive either 50 mg/day of diosgenin-rich yam extract (*n*=18) or placebo (*n*=13) [87]. Those who received yam extract saw a significant age-dependent increase in the Repeatable Battery for the Assessment of Neuropsychological Status total score compared to placebo, with older subjects (>47 years of age) achieving greater improvement. On the Repeatable Battery for the Assessment of Neuropsychological Status subtests, semantic fluency significantly improved in the yam extract group compared to placebo. Therefore, diosgenin yam extract was able to improve cognitive function in adults over a wide range of ages, but with more pronounced effects occurring in individuals over 47 years of age. However, a mechanistic explanation for diosgenin-rich yam’s effect remains unknown, as the relationship between morphological changes in the brain and cognitive function in neurodegenerative disease progression was not explored.

#### 3.2.12. Withania somnifera (L.) Dunal - Ashwagandha

*Withania somnifera*, commonly known as ashwagandha, is an Ayurvedic plant that has been used medicinally for over 3,000 years [239]. It is an adaptogen that helps the body respond more effectively and resiliently to stress, promotes immunity, and combats oxidative stress and cellular damage due to its antioxidant properties. The bioactive C28-steroidal lactones found in the leaves of *Withania somnifera* (such as Withaferin A and sitoindosides VII-X) have been shown to have antioxidant, anti-inflammatory, anxiolytic, and neuroprotective properties. In addition, the roots of *Withania somnifera* contain Withanoside IV and its metabolite sominone, which have been demonstrated to induce neuronal outgrowth and synaptogenesis. Furthermore, the plant has been shown to inhibit AChE and protect against cognitive impairment in rats [240].

In an 8-week randomized, double-blind, placebo-controlled, parallel-group trial, the effect of 300 mg twice/day of ashwagandha root extract (KSM-66 Ashwagandha; *n*=25) versus placebo (*n*=25) in adults aged 35+ with MCI was assessed [88]. The KSM-66 Ashwagandha was a 100% aqueous extract of the roots of *Withania somnifera* and contained 5% withanolides. Subjects were evaluated at baseline, 4 weeks, and 8 weeks on memory, visuospatial capabilities, executive function, and attention. The WMS-III was used to assess immediate, general, and working memory. In addition, WMS-III Visual Reproduction I and II subtests were used to assess visual memory, and the Shepard Mental Rotation Task was used to assess the subject’s cognitive rate of spatial processing. The Wisconsin Card Sort Test and Eriksen Flanker Task were also used to assess the subjects’ executive function. Finally, TMT A and the Mackworth Clock Test were used to assess attention and information-processing speed. The treatment group performed significantly better on the tests that assessed immediate and general memory, executive function, attention, and information-processing speed after 8 weeks compared to the placebo group. However, the effects of treatment on working memory and visuospatial processing were inconclusive, as the difference in performance between the 2 groups during these tasks was insignificant. No adverse effects were reported in the study, deeming the treatment tolerable. This study demonstrates that *Withania somnifera* offers promise as a safe and effective treatment for improving immediate and general memory, executive function, attention, and information-processing speed in patients with MCI.

In another randomized, double-blind, and placebo-controlled study of subjects with bipolar disorder (aged 18-65; *n*=60), *Withania somnifera* extract was assessed for its effect on cognitive dysfunction [89]. Subjects were randomly assigned for 8 weeks to either 500 mg/day of *Withania somnifera* in the form of a tablet called Sensoril, which contains a minimum of 8% withanolides, 32% oligosaccharides, and a maximum of 2% Withaferin A (*n*=30) or placebo (*n*=30) and were tested at baseline and at post-intervention. The subjects continued taking their medications as usual. The Set Shifting Test, Strategic Target Detection Test, Flanker Test, Auditory Digit Span, Word List Memory, and Finger Tapping Test were used to evaluate executive function, processing speed, attention, working memory, memory, and psychomotor speed, while the Penn Emotional Acuity Test was used to evaluate social cognition. Subjects in the treatment group performed significantly better on the Flanker Test (neutral mean response time), Auditory Digit Span (mean digit span backward), and Penn Emotional Acuity Test (mean social cognition response rating) compared to placebo at the end of the study. No other cognition tests differed significantly between the treatment and placebo groups. Adverse effects were mild, with no difference between the treatment and placebo groups. These results indicate that *Withania somnifera* extract may safely improve aspects of cognition such as verbal working memory, response time, and social cognition response in subjects with bipolar disorder. However, the effect of *Withania somnifera* extract on other aspects of cognition is inconclusive and warrants further investigation.

#### 3.2.13. Xanthines

Antioxidant xanthines are abundant in natural sources (e.g., tea, coffee, and chocolate) in molecular forms such as caffeine (1,3,7-trimethylxanthine) [241]. Caffeine is widely consumed for its adenosine antagonistic effects, which provide excitation and CNS stimulation [242] by modulating neurotransmitter release (e.g., dopamine, acetylcholine, norepinephrine, and serotonin) [243], thus altering attention and mood [244]. Therefore, studies have found that caffeine is successful in improving cognition by improving alertness [245], reducing reaction times [246], improving concentration and accuracy [247], and even enhancing short-term memory [248]. Thus, many studies have investigated the acute effects of coffee consumption on cognition.

Forty-three regular caffeine users were randomized to receive either caffeinated (~50 mg caffeine) or decaffeinated coffee before performing several cognitive tests [90]. The treatments were offered at 2 time points, 1 week apart. Those who had caffeine had a significantly faster mean reaction time on the Stroop Test and performed significantly better on the Jansari Assessment of Executive Functions (average performance, planning, creative thinking, event-based prospective memory, time-based prospective memory, and action-based prospective memory). Therefore, caffeine appeared to successfully improve executive functioning and Stroop reaction time.

In a similar study of healthy adults, 128 subjects were randomized to a 1-time consumption of either caffeinated (65 mg; *n*=64) or decaffeinated coffee (*n*=64) before performing cognitive tasks [91]. Those who received caffeine had significantly faster simple reaction times, indicating increases in speed of encoding and response to a novel stimulus. However, caffeine exerted no significant effects on working memory tasks. Interestingly, caffeine had differential effects on extroverts and introverts, as extroverts performed better after caffeine in the running memory task, and introverts performed worse. Similarly, the effect of caffeine intake and trait anxiety interacted, as caffeine improved performance in the mental rotation task for those with high anxiety and hindered performance in those with low anxiety. Therefore, the beneficial effects of caffeine not only depended on the nature of the task (improving reaction time rather than working memory), but also the nature of the individual (more effective in extroverts and high-anxiety individuals for certain tasks).

In a crossover study, 72 young adult regular coffee drinkers were randomized to a 1-time consumption of either caffeinated (100 mg) coffee, decaffeinated coffee, or coffee-flavored placebo water at each of 3 study visits before performing cognitive tests [92]. The regular coffee group had significantly better digit vigilance accuracy and reaction time, compared to the decaffeinated coffee and placebo groups, respectively. The regular coffee group also had significantly faster RVIP reaction time compared to the placebo group. Both caffeinated and decaffeinated coffee significantly improved subjective feeling of alertness, compared to placebo, and caffeinated coffee decreased feelings of tiredness and headaches compared to both other groups. Overall mood and mental fatigue ratings were also significantly better for the regular coffee group compared to placebo. However, regular coffee increased feelings of jitteriness compared to placebo in young females and compared to decaffeinated coffee in older males. Therefore, caffeine increased reaction time and accuracy, enhanced alertness and overall mood, and mitigated feelings of tiredness, headaches, and mental fatigue. However, these positive findings were not without feelings of jitteriness, although age and sex appeared to influence the extent of jitters.

In addition to improvements in performance, caffeine has also demonstrated physical neuroprotective effects during cognitive tasks. Changes in regional cerebral blood volume have occurred in the frontal region of the brain during cognitive tasks [249], but it is unknown whether they occur specifically in the associated area in the prefrontal cortex, which is related to mental work [250]. Caffeine has been found to constrict blood vessels in the brain, explaining its ability to reduce headaches [249], so it may also reduce regional cerebral blood volume.

In a crossover study, 14 subjects were randomized to 1-time consumption of either caffeinated (180 mg) or decaffeinated coffee and then consumed the other beverage after at least 1 week. After each consumption, subjects performed the Uchida-Kraepelin psychodiagnostics test to compare the effects of mental work on prefrontal cortex regional cerebral blood volume with and without caffeine [93]. On average, the number of answers per session increased in the caffeine group and decreased in the decaffeinated group, likely due to mental fatigue. Regional cerebral blood volume in the inferior frontal cortex increased with each mental work task, though this occurred to the same degree before and after caffeine intake. Thus, improvements in performance with caffeine were not reflected in the regional cerebral blood volume in the inferior prefrontal cortex. However, in the caffeine group, regional cerebral blood volume decreased during rest periods between tasks due to vasoconstriction. This indicates that in addition to caffeine’s ability to activate prefrontal cortex neurons to improve cognition, the vasoconstrictive capacity of caffeine protects against potential hyperemia induced by mental work. Overall, the beneficial effects of caffeine on cognitive function seem to clearly extend far beyond its properties as a stimulant.

While both coffee and tea contain caffeine, the effects of tea on cognition and human performance are less widely studied. While coffee and tea, when matched for caffeine, have similar effects on alertness, tea has been found to produce more consistent levels of arousal throughout the day [251]. This could be due to the components of tea, such as flavonoids or theanine, that coffee lacks. When ingested alone, theanine has been found to induce calmness and synchronicity of brain activity [252], but when combined with caffeine it induces even greater synchronization [253], providing improvements in attention [254].

Therefore, in a crossover study, 30 habitual caffeine drinkers were randomized to receive either caffeinated black leaf tea (either 37.5 mg/1 cup or 75 mg/2 cups), caffeinated black coffee (75 mg/1 cup or 150 mg/2 cups), or bottled water at 4 different time points throughout the day before performing cognitive tasks to compare the acute effects of coffee and tea on cognitive and psychomotor performance [94]. For the critical flicker fusion task, the analysis revealed that water was associated with decreased performance over time, while the caffeinated beverages maintained performance throughout the day, with the best results shown for 1 cup of tea. After the first drink, a significant interaction between treatment and time was found, as caffeine maintained alertness, while water was associated with a decline. Furthermore, at the same dose of caffeine, tea produced a rapid increase in the critical flicker fusion threshold between 30 and 90 min post-consumption compared to coffee. Performance did not differ significantly between doses. For the line analogue rating scales for sedation, 1 cup of tea was associated with higher subjective feelings of alertness throughout the day, which is consistent with its effects on critical flicker fusion performance. Only the first drink influenced the line analogue rating scales for sedation, with significantly higher levels of alertness for caffeinated beverages compared to water. The second drink showed a significant treatment effect for the recognition reaction time component of the choice reaction time task, where water slowed reaction time and the caffeinated beverages maintained baseline performance. At the same caffeine dose, compared to tea, coffee was associated with significantly faster reaction times between 10 and 90 min post-consumption. However, caffeine had an acute quadratic effect following the first drink (1 cup was superior for reaction time) and a linear dose-response effect following the third drink (2 cups slowed reaction time). Therefore, compared to water, caffeine had a significant acute effect on arousal, particularly at lower doses. Furthermore, at the same caffeine dose, tea and coffee are superior at acutely enhancing performance for different tasks. Tea improved critical flicker fusion (a measure of alertness), while coffee improved choice reaction time (a measure of reaction time), but the effects of coffee on reaction time were not stable. Therefore, the exact differential effects of tea and coffee on cognition remain unclear and warrant further investigation.

Additional studies have been performed on the effects of tea on cognition. In a 2-part crossover study, participants were randomized in study 1 (*n*=26) to receive 2 servings of either black tea (50 mg caffeine and 23 mg theanine/serving) or placebo tea over 60 min, and in study 2 (*n*=32) they received either 3 servings of black tea (30 mg caffeine and 12 mg theanine/serving) or placebo tea over 90 min before performing cognitive tasks [95]. In study 1, those who received black tea had more correct responses on intersensory attention subtasks (auditory and visual) and responded faster (visual) compared to placebo. They also had more correct responses for task switching and felt significantly more alert but less calm compared to placebo. In study 2, the number of correct responses or reaction times for intersensory attention did not differ between groups, but those who received black tea had more correct responses for task switching, felt significantly more alert, and trended toward greater feelings of contentedness compared to placebo, with no difference in feelings of calmness. Therefore, both studies showed that black tea improved overall attention for the intersensory attention tests and accuracy for novel stimuli on the task-switching tests. The greater improvements in reaction time and accuracy and enhanced measures of self-reported mental state occurred with the stronger tea in study 1, likely due to the combined effects of caffeine and theanine.

In another study, 23 subjects were randomized to receive either matcha tea, a matcha snack bar (each containing 4 g matcha powder with 67 mg theanine and 136 mg caffeine), placebo tea, or placebo snack bar, while performing cognitive tasks at both baseline and post-consumption [96]. The treatments were offered for 4 days, and each day cognitive function was assessed before and after consumption. Response speed for the simple reaction time task was faster in those who received matcha tea compared to the placebo drink. For choice reaction time, those who received either type of matcha had significantly improved response speeds compared to either placebo. For the speed of attention factor, the interaction between format and condition was significant, although the significance disappeared using multiple pairwise comparisons. For delayed picture recognition accuracy, spatial working memory accuracy, and the working memory factor, both drink formats yielded better accuracy compared to the bar format. Therefore, consumption of matcha in both tea and bar forms slightly improved cognitive performance on a few specific attentional tasks, and the drink form outperformed the bar form on accuracy of delayed picture recognition and spatial working memory. Both forms consistently affected attention abilities but had more significant effects on working memory. These results appear more similar to the effects of caffeine alone, rather than the synergistic effects of caffeine and theanine, suggesting further research is warranted.

Xanthines are also found in chocolate; in addition to caffeine, cocoa products have an extremely high concentration of a metabolite of caffeine called theobromine (3-7-dimethylxanthine) [255]. The effects of theobromine on the CNS have been found to be weak, but in large doses, it has been found to cause increases in energy, motivation, and alertness in some individuals [256].

In a 2-part study, the effects of chocolate consumption on cognition were investigated [97]. In the first study, 20 individuals were randomized to 1-time consumption of either chocolate with cocoa powder, chocolate containing 250 mg theobromine and 19 mg caffeine, or a placebo. The cocoa powder and caffeine groups had significantly faster simple reaction time response speeds, and the caffeine group had significantly better performance on the RVIP task compared to placebo with cocoa powder marginally better. The cocoa powder and caffeine groups also had significantly higher self-reported energetic arousal, and the caffeine group had significantly improved hedonic tone (cocoa powder was marginally better) compared to placebo. Feelings of hunger decreased only after cocoa powder consumption, but not caffeine, compared to placebo, and headache symptoms decreased only after caffeine, but not after cocoa powder. Therefore, the cocoa powder and caffeine treatments showed similar effects, although cocoa powder was slightly less effective. In the second study, 22 individuals were randomized to 1-time consumption of 3 different treatments with varying levels of methylxanthines (i.e., caffeine and theobromine): zero methylxanthine, low methylxanthine (8 mg caffeine and 100 mg theobromine), and high methylxanthine (20 mg caffeine and 250 mg theobromine) or placebo (water). These treatments match the methylxanthine contents of white, milk, and dark chocolate, respectively. High methylxanthine significantly increased reaction speed compared to zero methylxanthine for the simple reaction time task, and both low and high methylxanthine improved RVIP performance compared to zero methylxanthine. Mood scores for energetic arousal and hedonic tone showed a similar effect to the simple reaction time and RVIP, with higher scores for methylxanthine-containing treatments. Therefore, the effects of milk and dark chocolate due to methylxanthine content do not appear to be significant. Overall, both studies exhibited some improvements in cognition and mood. Each treatment arm contained around or below the lower range of caffeine doses found to have detectable stimulant effects, suggesting that these psychoactive changes were likely due to the methylxanthine content.

In another study, subjects were randomized to receive either dark chocolate (26.8 mg caffeine and 197.5 mg theobromine/day; *n*=10) or white chocolate (non-detectable methylxanthine; *n*=8) daily for 30 days to investigate the effects of methylxanthine-rich chocolate intake on cognition [98]. Cognitive function was measured at baseline, after 30 days, and 3 weeks after the end of the intervention. Dark chocolate intake resulted in a marginally higher number of correct answers on the modified Stroop Test at 30 days compared to baseline, while white chocolate intake had no impact. These numbers remained marginally higher at follow-up in the dark chocolate group compared to the white chocolate group, and the dark chocolate group performed better overall. For the digital cancellation test (3 trials), dark chocolate significantly increased total performance numbers at 30 days compared to baseline for trial 3 (but not trials 1 or 2), whereas white chocolate had no effect. Total performance in the dark chocolate group remained significantly higher compared to the white chocolate group at follow-up. However, omission ratio significantly decreased at 30 days in trials 1 and 2 for the dark chocolate group compared to baseline. The NGF level increased at 30 days for the dark chocolate group but returned to the baseline value at follow-up. In the white chocolate group, NGF did not change at all. Plasma theobromine concentration increased significantly throughout the intervention period in the dark chocolate group, returning to baseline at follow-up, whereas it did not change in the white chocolate group. However, plasma caffeine levels did not change in either group at any point. Therefore, 1 month of dark chocolate consumption yielded a transient increase in NGF and plasma theobromine, which may promote more lasting neuronal plasticity and subsequent improvement in cognition.

Thus, these results using coffee, tea, and chocolate as sources of various xanthines are promising for improving cognitive function. In addition, these foods may have other naturally occurring phytochemicals that interact with the xanthines to produce positive cognitive effects.

### 4. Discussion

With the rising burden of chronic illnesses, the increasing morbidity and mortality of AD and other neurocognitive disorders have spurred great interest in brain health, both among medical providers and the public. In part due to a lack of effective treatment or preventive options, improvement of cognitive function using various dietary supplements has emerged as a particular area of focus. At present, the FDA-approved medications for the treatment of dementia can only briefly slow cognitive decline, while also causing significant adverse effects, and robust evidence for effective preventive strategies is also lacking [2]. Although regular physical exercise and a healthy dietary pattern that avoids nutritional deficiency currently form the basis for prevention of neurodegenerative disease [257], targeted nutritional supplementation tailored to the health status of the individual patient may offer additional benefit in the prevention and management of cognitive decline.

This narrative review of clinical trial data suggests that certain nutrients and phytonutrients are capable of significantly modulating cognition in different adult populations with varying health conditions that cause cognitive impairment. The populations in this review included AD and MCI, MS, traumatic brain injury, stroke, psychotic disorders, and healthy adults.

In AD and MCI, numerous nutrients and phytonutrients have been studied for their effects on cognitive function. A reduced rate of cognitive decline was exhibited in trials using α-lipoic acid as an adjunct treatment to traditional medications [3,4]. Likewise, *Bacopa monnieri* has shown improvements in some aspects of cognitive function, perhaps due to anti-inflammatory and antioxidant effects [35,36]. In contrast, B vitamin supplementation in AD was not shown to improve cognitive function or delay decline [11]. However, in patients with MCI and high homocysteine [14], B vitamin treatment may slow the decline in cognitive function, suggesting that the effects of B vitamin treatment may depend on the baseline nutritional status. Cholinergic precursors including choline, lecithin, and phosphatidylserine have been studied in several trials of individuals with AD, MCI, or age-related cognitive impairment with inconsistent results [15,18-21]. Overall, the reviewed studies indicate that cholinergic precursors may improve some aspects of cognitive function without adverse effects, although methodological issues and heterogeneity of the studies make definite conclusions about benefit impossible at this time. Vitamin D has shown promise in AD and MCI, where improvements in cognitive function were noted, along with improvements in blood lipids and Ab-related biomarkers [23,24]. On the other hand, vitamin E has shown inconsistent results. Some studies have indicated that vitamin E may modestly delay the progression of dementia [6], but another study raised the question of differential effects in responders and non-responders [7]. Responders with a reduction in oxidative stress saw maintenance of cognitive function, while non-responders who showed no reduction in oxidative stress actually saw a decrease in cognitive function, possibly through vitamin E acting as a pro-oxidant [7]. *Ginkgo biloba* is an herb that has been widely studied with conflicting results. Some research showed promise through modest improvements in various aspects of cognitive function [42,43,49,51-53,55,57], although the effects were typically small and not always clinically significant. The design and quality of studies were also variable, with some trials lacking blinding or placebo control, and some experiencing significant attrition. Other research showed no significant effect of *Ginkgo biloba* after treatment [48,50]. American and *Panax ginseng* have also been evaluated for their effects on cognitive impairment. As with *Ginkgo biloba*, ginseng has shown modest improvements in some facets of cognition [59-61,63], while being limited by similar methodological concerns. Lion’s mane mushroom demonstrated improvement in cognitive function in older Japanese adults with MCI, with the improvement increasing over the course of 16 weeks and declining after 4 weeks of washout [66]. Ω-3 fatty acids have also been studied with inconsistent results. While some research showed modest improvement in clinical status and an attenuated decline in cognitive ability [5,27], possibly through reductions in inflammation and oxidative stress, another study did not reveal any benefit in the prevention or treatment of AD [159]. A less studied compound is aloe polymannose, a polysaccharide that showed statistically and clinically significant improvements in cognition and multiple immune and inflammatory markers in an open-label study [33], warranting larger controlled studies to further investigate its potential for cognitive enhancement. Tart cherry juice may also modestly improve cognitive function after chronic consumption [80], while a similar acute improvement in cognition has not been observed [81]. Saffron has shown promise in patients with varying degrees of cognitive impairment, and small studies suggest it may offer benefits comparable to AChEI medications [76-79]. Finally, in a study, *Withania somnifera* demonstrated promising results as a safe and effective treatment for improving immediate and general memory, executive function, attention, and information-processing speed in patients with MCI, but was inconclusive in its effect on working memory and visuospatial processing [88]. Overall, in AD and MCI, numerous dietary supplements and plant extracts have shown promise in individual trials for improving cognitive functioning. However, the totality of evidence for any one nutrient or phytonutrient is inconsistent at best, and larger-scale trials with better methodological designs are needed to make definitive recommendations about their use.

Nutrients and phytonutrients have also been evaluated in psychotic disorders including schizophrenia and psychosis. A very small study in subjects with EEG similarities to those with schizophrenia examined the effect of citicoline supplementation with preliminary findings of improved executive function [17]. American ginseng has also been studied with promising results, showing improvement in working memory and reduction in extrapyramidal effects, which often greatly impair quality of life and functioning in patients with schizophrenia [65]. In a study, NAC was also able to improve multiple cognitive symptoms (excluding positive symptoms) found in schizophrenia [8], while another study using NAC found an improvement in working memory in patients with psychosis after 24 weeks of supplementation [9]. *Withania somnifera* showed significant improvements in neutral mean response time, mean digit span backward, and mean social cognition response rating in subjects with bipolar disorder, but not other aspects of cognition [89]. Overall, nutrients and phytonutrients have rarely been evaluated for their effects on cognition in this patient population, but the results of these few studies warrant further investigation in the treatment of psychotic disorders, where conventional medical treatment is typically lacking in efficacy.

In MS, an aloe polysaccharide multinutrient supplement was assessed in a small trial, showing improved cognition, mood, and functioning symptoms and quality of life over the course of the 12-month intervention [34]. Vitamin D3 supplementation was also found to improve cognitive performance in 25(OH)D deficient patients with relapse-remitting MS [22], while a *Ginkgo biloba* extract did not improve cognitive function over a 12-week period [54]. Once again, few nutrients and phytonutrients have been studied for their effects on cognitive functioning in this patient population.

Although nutrients and phytonutrients have most often shown benefit in chronic disorders, acute conditions may also improve from their use. In particular, NAC appears to be beneficial in the treatment of cognitive symptoms of blast exposure-induced mTBI, with earlier administration leading to improved outcomes [10]. The risk of cognitive dysfunction after stroke is elevated, so treatments to lessen post-stroke complications are needed. Citicoline has shown promise in improving some domains of cognitive function over 12 months of treatment after stroke without any noted significant adverse effects [16].

In addition to various diseases and disorders, nutrients and phytonutrients have been examined for their efficacy on cognitive function in healthy adults. For example, *Bacopa monnieri* has been studied in a few instances in healthy populations of various ages and shows promise in improving several domains of cognitive function with minimal recorded adverse effects [35,37-40]. Lion’s mane also showed a significant improvement in cognitive function in older adults after 12 weeks of consumption [67]. Furthermore, *Rhodiola rosea* has shown hopeful results in improving mental capacity for work, fatigue, anxiety, mood, and cognitive performance in healthy adults [194-198]. However, several vitamins have thus far failed to show consistent benefits. In adults with elevated homocysteine but intact cognitive function, B vitamin supplementation has been inconsistent in improving cognition [12,13]. Likewise, vitamin D supplementation does not appear beneficial for improving cognition in healthy adults, although those with the lower baseline 25(OH)D levels may experience some improvement in nonverbal memory [25,26]. The effects of zinc supplementation on cognition are similarly very limited [28]. *Ginkgo biloba* has also been studied in healthy adults with conflicting results. Some research has shown no benefit [48], while other studies suggest modest benefits on specific cognitive domains [43,45-47]. Working memory appears to be enhanced by both American and *Panax ginseng* [58,62,64]. Supplementation with aloe polysaccharides has shown memory benefits [30,32], although the results are equivocal [31].

Some plant extracts well known in culinary use have also been studied for their potential cognitive benefits in healthy individuals. Rosemary has been evaluated as an oral supplement and in aromatherapy with largely underwhelming results. Modest improvements in cognition and mood were noted in some studies, although others showed either no significant effects or negative effects on performance with elevated doses [72-75]. However, curcumin showed more promise in several studies, showing improvements in cognition and attention [83-86], while yam extract was able to improve measures of cognitive performance with more pronounced effects in older individuals [87]. The effect on cognition of xanthines, antioxidants found in foods such as coffee, tea, and chocolate, has also received considerable research interest. Various studies suggest that xanthines such as caffeine may improve cognition, including alertness, accuracy, and reaction time [90-98]. Thus, several plant extracts or components have shown promise in improving cognitive function among healthy adults, although the evidence stems largely from small trials with various methodological differences and limitations.

Overall, the reviewed clinical trials have displayed a wide range of results on cognitive function, going from negative (high doses of rosemary) to null (several vitamins) to promising (aloe polysaccharides, *Bacopa monnieri*, curcumin, *Rhodiola rosea*, saffron, *Withania somnifera*, and xanthines). The nutrients and phytonutrients showing promise in improving cognitive function certainly warrant continued study. These primarily supplemental products have the potential to serve as relatively inexpensive preventive and treatment strategies for cognition, and encouragingly almost all of these nutrients and phytonutrients were well-tolerated with minimal documented adverse effects. In addition, many of them, e.g., caffeine, curcumin, and saffron, have a long history of culinary use and are found in plants that are known to be healthful, adding to the safety profile of long-term consumption. However, amounts greater than those used in food preparation should continue to be evaluated for safety where they are not already well-documented, e.g., rosemary in supraphysiological doses revealed negative effects on cognitive performance, but no serious adverse events were recorded [72].

Trying to generalize the results of such a large number of articles on 21 nutrients and phytonutrients is not simple. The heterogeneity of study design and methodological quality pose primary limitations to generalizability. While many of the studies were randomized, double-blind, placebo-controlled trials, others were limited by small samples and lack of blinding, placebo, or control group. Treatment amount and type and follow-up time across studies also differed significantly, making it difficult to predict any long-term risks for certain nutrients and perhaps preventing the detection of benefits that may only manifest with ingestion of a nutrient or phytonutrient for years. Cognition is assessed with a multitude of neuropsychological tests, so it is difficult to determine how, e.g., changes on the ADAS-cog compare to changes on the many versions of the WAIS. Most of these studies tested only 1 nutrient or phytonutrient, but across the board they did not include dietary analysis of the subjects, so it is unknown if and how food intake and nutritional status could have affected the results, particularly for the vitamins and minerals included in this review. Bioavailability and absorption of the active ingredients studied are additional key considerations for assessing the clinical outcomes. For example, curcuminoids are known to have very low naturally occurring bioavailability and absorption, and the way they are formulated in a test product could have dramatic effects on study outcomes [231]. Sun exposure should be measured for any study assessing supplemental vitamin D consumption but was not included in the study design of those studies. Every growing season has the potential to modify the nutrient profile of a plant, so its levels of active ingredients in a supplemental product could subsequently be affected, which ultimately impacts its clinical efficacy. Due to these limitations, additional large, well-controlled studies are needed to investigate the safety and efficacy of these nutrients and phytonutrients in the context of treating and preventing various neurocognitive disorders.

This type of narrative review has several inherent limitations. We did not synthesize or pool data for each nutrient or phytonutrient and disease or disorder. Thus, it is difficult to make any conclusions about the overall efficacy of these nutrients and phytonutrients. Furthermore, we did not compare the efficacy of different dosages among studies, so in most cases, the ideal dose of any nutrient or phytonutrient is not clear. It is also not certain how dosages may need to differ depending on the disease or disorder. In addition, we did not limit our review to a certain treatment duration, so it is difficult to determine how outcomes translate to improvements in lifespan, even for studies that evaluated a year-long intervention.

In summary, this narrative review highlights the current evidence of specific nutrients and phytonutrients that may improve aspects of cognitive function, yielding improved performance on key cognitive tests and perceived attentiveness and affect. Thus, many of these nutrients may have potential benefits in the prevention and adjunct treatment of conditions characterized by cognitive dysfunction, such as AD and other related neurodegenerative disorders. It should be noted that certain individual nutrients and phytonutrients appear more promising in improving cognitive function, and treatment efficacy may differ depending on disease state, so treatment regimens should be individualized as much as possible with ongoing evaluation of benefits and risks for adjustment. Dietary supplements also may interact with some prescription and over-the-counter medications through direct interaction or modulation of key enzymes implicated in drug metabolism, warranting additional caution when recommending their use. Plant components also display immensely complex multi-physiological activity and often act synergistically, making the function of isolated extracts less predictable. Thus, thoughtful review of current medication and dietary supplement intake should precede the addition of new nutrients and phytonutrients, especially among the elderly who are more vulnerable to adverse interactions through polypharmacy and changes in drug metabolism. As additional research is necessary to make robust recommendations for any nutrient or phytonutrient, some of those that were reviewed, e.g., aloe polysaccharides, *Bacopa monnieri*, *Ginkgo biloba*, and *Rhodiola rosea*, may offer an avenue for improving cognitive function in illnesses currently lacking effective conventional treatment.

### Conflict of Interest

The authors declare no conflicts of interest.
